# Small non-coding RNAs encoded by RNA viruses: old controversies and new lessons from the COVID-19 pandemic

**DOI:** 10.3389/fgene.2023.1216890

**Published:** 2023-06-21

**Authors:** Carolina Ruivinho, Margarida Gama-Carvalho

**Affiliations:** BioISI—Biosystems & Integrative Sciences Institute, Faculty of Sciences, University of Lisboa, Lisboa, Portugal

**Keywords:** RNA virus, viral miRNAs, sncRNAs, non-coding RNAs, host-pathogen interaction

## Abstract

The recurring outbreaks caused by emerging RNA viruses have fostered an increased interest in the research of the mechanisms that regulate viral life cycles and the pathological outcomes associated with infections. Although interactions at the protein level are well-studied, interactions mediated by RNA molecules are less explored. RNA viruses can encode small non-coding RNAs molecules (sncRNAs), including viral miRNAs (v-miRNAs), that play important roles in modulating host immune responses and viral replication by targeting viral or host transcripts. Starting from the analysis of public databases compiling the known repertoire of viral ncRNA molecules and the evolution of publications and research interests on this topic in the wake of the COVID-19 pandemic, we provide an updated view on the current knowledge on viral sncRNAs, with a focus on v-miRNAs encoded by RNA viruses, and their mechanisms of action. We also discuss the potential of these molecules as diagnostic and prognostic biomarkers for viral infections and the development of antiviral therapies targeting v-miRNAs. This review emphasizes the importance of continued research efforts to characterize sncRNAs encoded by RNA viruses, identifies the most relevant pitfalls in the study of these molecules, and highlights the paradigm changes that have occurred in the last few years regarding their biogenesis, prevalence and functional relevance in the context of host-pathogen interactions.

## 1 Introduction

Most organisms, including bacteria, blue-green algae, fungi, plants, insects, and vertebrates are known to be infected by viruses. Although the first written record of a human virus disease dates to ancient Egypt, significant milestones in virus research are all from the 20th century. Following the demonstration of viruses as filterable infectious particles in 1892, advances on electron microscopy and molecular composition analysis created new opportunities for discovery, with modern biotechnology and genomics supporting the current understanding of replication mechanisms and virus-host interactions (Ryu, 2017).

Socioeconomic and environmental changes have increased the frequency and impact of viral outbreaks in recent decades, as recently seen for Zika virus (ZIKV), chikungunya virus (CHIKV), Ebola virus (EBOV), and the global COVID-19 pandemic caused by the severe acute respiratory syndrome coronavirus 2 (SARS-CoV-2) (Li et al., 2005; Devaux, 2012; Holmes et al., 2016; Metsky et al., 2017). In counterweight, science is advancing at a rapid pace, supporting a faster characterization and better control of the viral agents responsible for these diseases. During the AIDS pandemic that started in 1981, it took 2 years to identify HIV-1 as the responsible pathogen, and almost 3 years more to provide efficient diagnostic tests based on serology (Sankaran and Weiss, 2021). However, when the first COVID-19 cases appeared in 2020, scientists obtained the full genetic sequence of the SARS-CoV-2 virus from a patient within 2 days and developed a reliable PCR test to detect infection in less than a week (Sankaran and Weiss, 2021).

**FIGURE 1:**
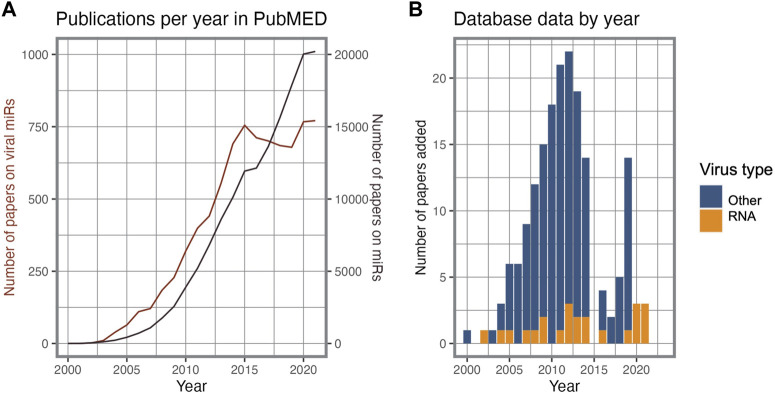
Research interest on viral miRs. **(A)**. Timeline of PubMED publications on miRs and viral miRs. **(B)**. Viral miR entry source per publication year and virus type in the VirBase database.

As obligate intracellular pathogens, viruses make extensive use of the host cell’s machinery to replicate, regulating host gene expression to enhance viral processes. The study of host-virus interactions has not only provided exceptional insights into the molecular mechanisms of pathogenesis, but also contributed to numerous discoveries in the field of cell biology (Welch, 2015). Molecular interactions mediated by proteins have been the subject of the majority of systematic studies of virus-host interactomes, whereas RNA-RNA interactions have only more recently attracted attention. The study of virus-derived sncRNAs (v-sncRNAs), including from RNA viruses, initially attracted great attention, but experienced an interim decline in interest compared to overall research on sncRNAs (Figure 1). This decline may be attributed to a combination of factors, including the technical challenges and limitations associated with studying these molecules and demonstrating their functional role. These difficulties spurred aggressive debates regarding the evolutionary and biological relevance of the use of sncRNA regulation by RNA virus genomes, further contributing to the decline in interest in the search for these elements (Cullen, 2010; Whisnant et al., 2013; Aguado and tenOever, 2018). More recently, the extensive study of the SARS-CoV-2 has once again put this topic under the spotlight.

The primary aim of this review is to present an up-to-date overview of the significance of RNA virus-derived sncRNAs and their implications in viral infection. By incorporating the latest findings following the COVID-19 pandemic, we aim to consolidate and organize the scattered information present in the literature, ultimately contributing to a comprehensive understanding of the biological relevance of these molecules. Moreover, we attempt to identify key knowledge gaps and unexplored areas that warrant further investigation, thus guiding future research endeavors and facilitating advancements in our understanding of viral pathogenesis and host-virus interactions.

### 1.1 An overview of RNA viruses

**FIGURE 2:**
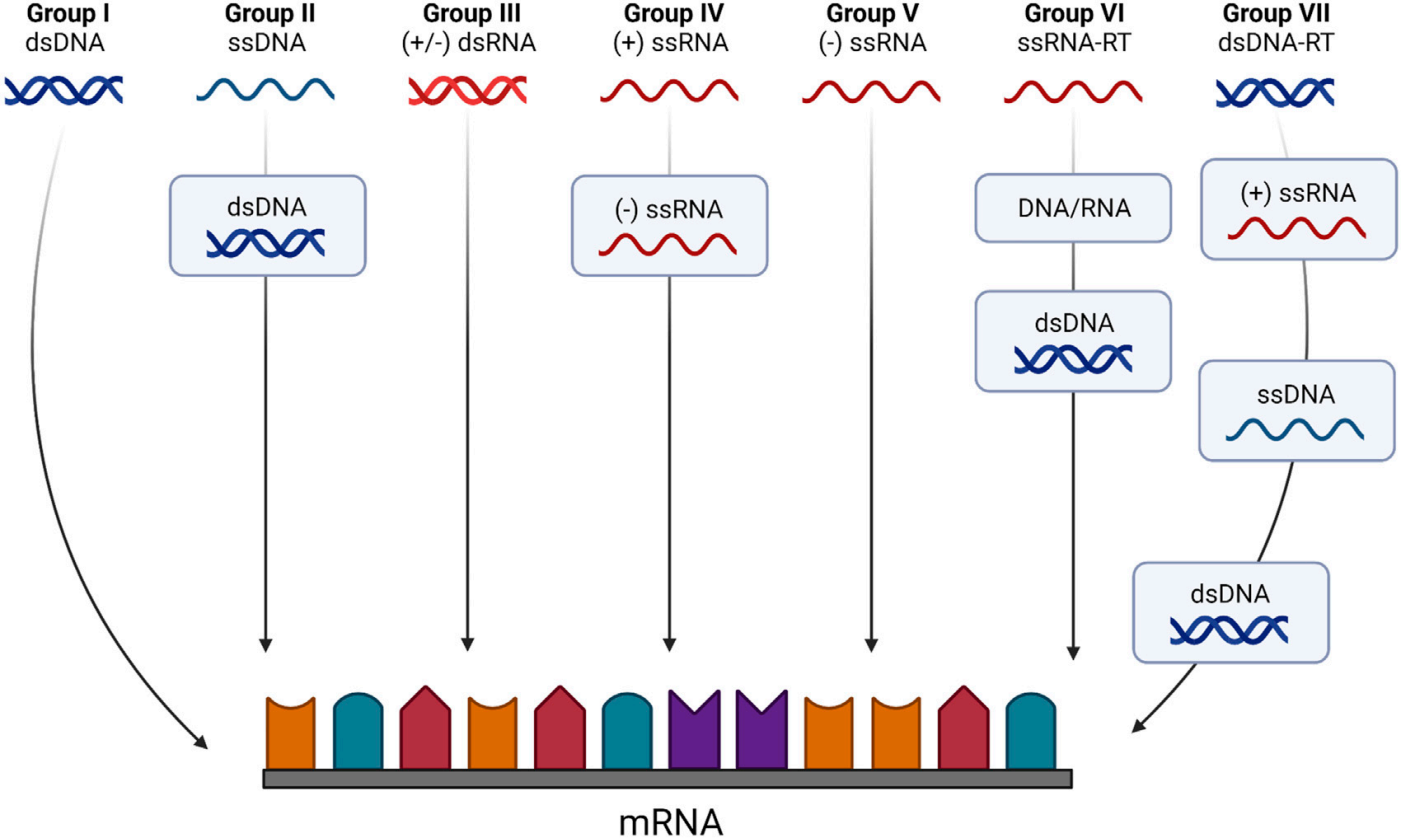
The Baltimore classification system based on genome type and replication strategy. According to this classification viruses belong to one of seven classes according to both their genome and the method used to generate mRNA molecules. Class I viruses are dsDNA viruses that can produce coding transcripts directly from genomic sequences. Class II viruses are ssDNA viruses that require the synthesis of a dsDNA intermediate to serve as template for transcription and mRNA synthesis. Classes III to V are strict RNA viruses. Class III viruses use an RNA-dependent RNA polymerase to transcribe mRNAs from their dsRNA genome directly, like the (−) sense ssRNA viruses that constitute Class V. Class IV viruses are (+) sense ssRNA viruses that require the synthesis of a (−) RNA intermediate to serve as a template for mRNA biogenesis. Classes VI and VII are viruses with ssRNA or dsDNA genomes, respectively, that require reverse transcriptase (RT) enzymes to produce an intermediary dsDNA that will serve as a template for viral gene expression. Created with BioRender.com.

Viruses have been classified based on their genetic material (Ryu, 2017)^.^ Beyond dividing viruses in two main groups based on their RNA or DNA genomes, the “Baltimore Classification” considers their mode of mRNA production, defining seven groups that denote different pathways from genome to mRNA production. Thus, DNA viruses are split between double-stranded (dsDNA; Group I, e.g., Herpesviridae) and single-stranded (ssDNA; Group II, e.g., Parvoviridae) genomes. Groups III to V correspond to RNA viruses with double-stranded (dsRNA; e.g., Reoviridae), single-stranded negative sense ([-]ssRNA; e.g., Orthomyxoviridae) or positive sense ([+]ssRNA; e.g., Flaviviridae) genomes, respectively. Some authors define the RNA virus family in a strict sense as composed by this group of viruses, which depend on virally encoded RNA-dependent RNA polymerase (RdRp) to replicate (Payne, 2017). However, retroviruses also have (+)ssRNA genomes, albeit replicating through DNA intermediates and relying on the host RNA polymerase for genome synthesis (Ryu, 2017). Given their dependence on the reverse transcriptase (RT) enzymes, retroviruses, together with the Hepadnaviridae family of DNA viruses that replicate through RNA intermediates, are considered as a separate class, corresponding to Baltimore classification groups VI and VII. The different groups of the Baltimore classification exhibit distinct characteristics and properties, each with their own replication strategies, namely, a fully cytoplasmatic *versus* nucleo-cytoplasmatic replication cycle, or the reliance on viral-encoded or host-encoded RNA polymerases for mRNA synthesis. These diverse properties have significant implications regarding the host-virus interactions that are established in the course of an infection and pose distinct challenges and opportunities in vaccine and therapy development. The schematic diagram in Figure 2 illustrates how each group of the Baltimore classification differently synthetizes their mRNAs.

Currently the NCBI virus database (Hatcher et al., 2017) contains genome sequence entries for 11,948 virus species, of which 4,187 (∼35%) are RNA genome viruses, divided by 57 families. As a result of their high mutation rates and frequent recombination events, RNA viruses are capable of rapidly adapting in response to environmental pressures such as the host immune response or antiviral drugs (Ryu, 2017). While these characteristics give RNA viruses a strong intrinsic pandemic potential, they also hinder the study of the complex biology of these viruses and complicate the development of effective vaccines and therapies for RNA viral diseases.

### 1.2 ncRNA elements in RNA genomes

**FIGURE 3:**
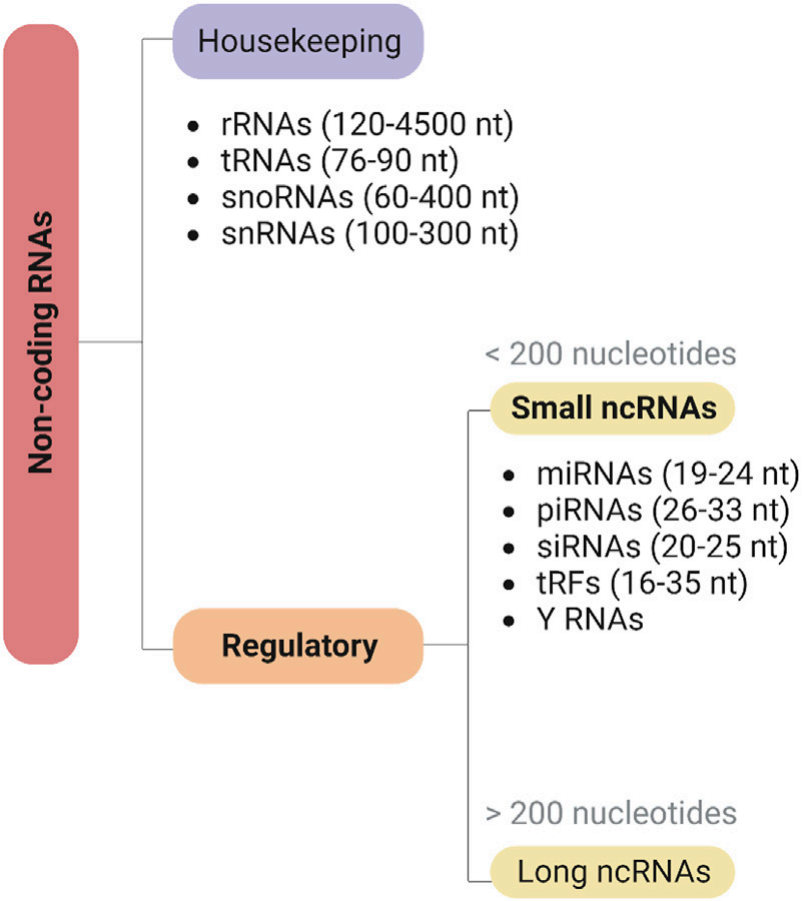
Classification of non-coding RNAs (ncRNAs). Noncoding RNAs can be classified into two main categories based on their biological functions: housekeeping and regulatory ncRNAs. Housekeeping ncRNAs, such as rRNA, tRNA, snoRNA, and snRNA, are involved in fundamental cellular processes. Regulatory ncRNAs, on the other hand, can be further divided into long (>200 nucleotides) or short (<200 nucleotides) ncRNAs, including miRNAs, piRNAs, siRNAs, tRFs, and Y RNAs, and play critical roles in gene regulation. Created with BioRender.com.

Since the discovery of regulatory ncRNAs, our understanding of gene regulation has been deeply transformed (Mattick and Makunin, 2006). ncRNAs can be classified according to their functional role as housekeeping or infrastructural ncRNAs, expressed in all cell types and carrying out essential functions in the cell, such as ribosomal RNAs (rRNAs), transfer RNAs (tRNAs), small nuclear RNAs (snRNAs), and small nucleolar RNAs (snoRNAs), or as regulatory ncRNAs (Mattick and Makunin, 2006) (Figure 3). Regulatory ncRNAs are divided into two broad categories according to their length: long non-coding RNA (>200 nucleotides; lncRNA) and sncRNAs (<200 nucleotides). The latter include microRNAs (miRNAs) and piwi-interacting RNAs (piRNAs), regulating post-transcriptional gene expression and the stability of mRNAs and transposons, respectively; short interfering RNAs (siRNAs), playing essential roles in the RNA interference (RNAi) pathway; and several other more recent or less studied classes, such as tRNA-derived fragments (tRFs) and Y RNAs (Shi et al., 2022). Thus, there is a wide array of RNA species that play important roles not only in normal cell function, but also as part of the cellular response to infection processes. Given the wide range of regulatory capacities displayed by these molecules, it is not surprising that viruses evolved to encode and utilize their own ncRNA elements (Withers et al., 2019). Indeed, despite the inability to encode proteins, multiple studies have demonstrated the importance of different types of ncRNA molecules in virtually all stages of the viral infection process, namely, in the regulation of viral replication, viral persistence, host immune evasion, and cellular transformation (He et al., 2008; Tycowski et al., 2015; Stamm and Lodmell, 2019; Talló-Parra et al., 2022).

Several virus families, covering most of the Baltimore classification scheme, have been shown to encode regulatory ncRNAs, in particular lncRNAs and miRNAs. Although DNA viruses are the foremost source of known viral ncRNAs, retroviruses and negative- and positive-strand RNA viruses also encode them. Currently, ViRBase (version 3.0), a database of interactions between viral and host ncRNAs contains 648 entries of viral encoded ncRNAs, of which 510 are miRNAs and 13 are long ncRNAs, encoded by 37 different viruses. It is worth pointing out that only nine of these are RNA viruses, seven of them causing human pathologies (Zika virus, Kunjin virus, Hepatitis C virus, Human immunodeficiency virus 1, Semliki Forest virus 4, Coxsackievirus B3 and SARS-CoV-2).

### 1.3 Viral miRNAs

miRNAs are a group of sncRNAs that range from 19 to 22 nucleotides in length (Bartel, 2018). These molecules act as fine-tuners of gene regulation, contributing to a large range of cellular processes, from differentiation to survival. The first miRNAs were identified in *Caenorhabditis elegans* in 1993 (Lee et al., 1993; Wightman et al., 1993), followed by the demonstration of their widespread presence and conservation across a very wide range of organisms (Bartel, 2018). These sncRNAs have been estimated to regulate approximately 60% of mammalian genes at the post-transcriptional level, with a single miRNA having the ability to regulate hundreds of mRNAs (Bartel, 2018). In comparison to protein-coding sequences, miRNAs display a higher level of cross-species conservation, indicating that they have been positively selected based on their regulatory function (Mahony et al., 2007). Many different regulatory pathways have been shown to be adversely affected by miRNAs, including metabolism, apoptosis, proliferation, differentiation and cancer (Bartel, 2018).

**FIGURE 4:**
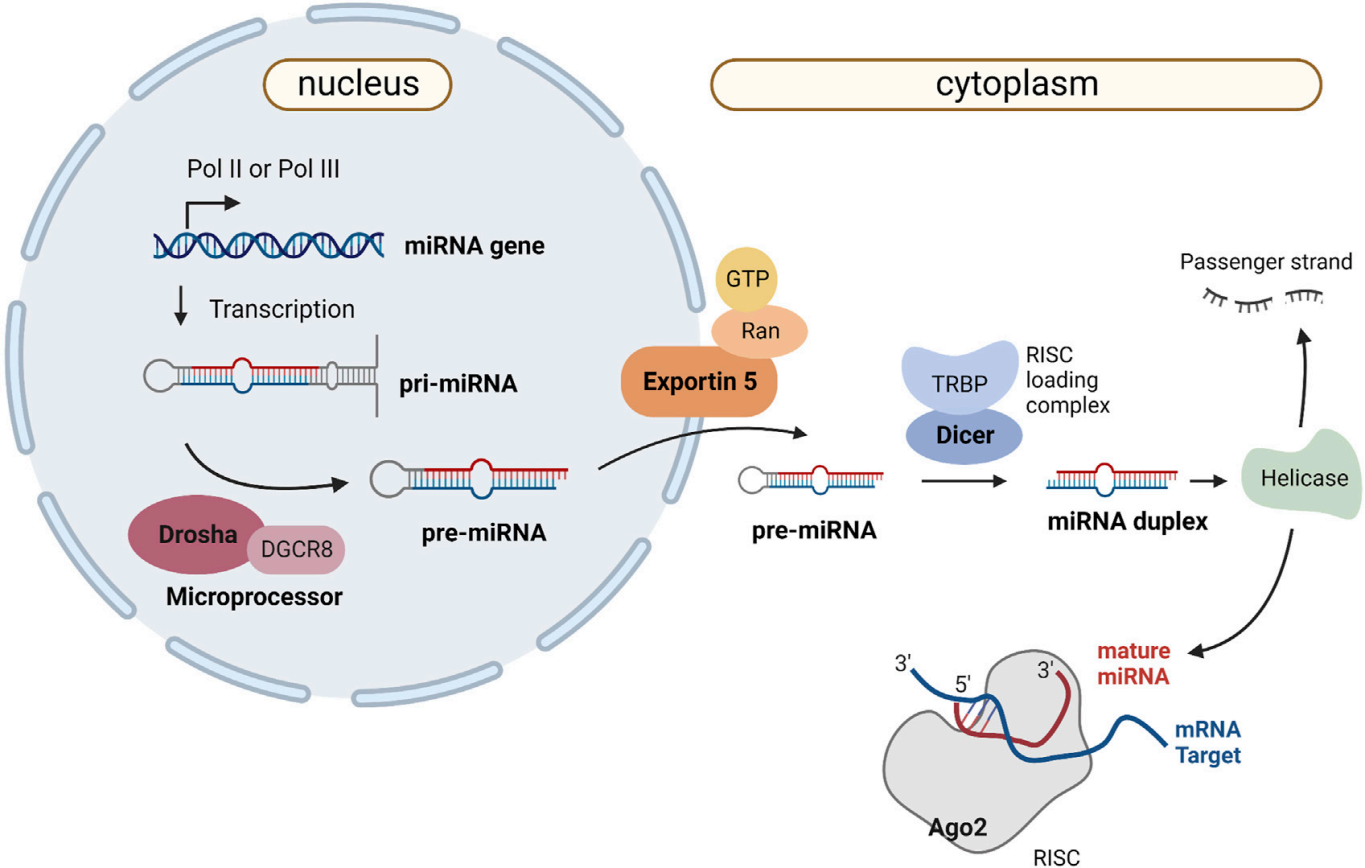
Canonical miRNA biogenesis and mechanism of action. Canonical miRNA biogenesis starts with RNA polymerase II or III (Pol II/Pol III) activity, which transcribes the primary miRNA (pri-miRNA) as a several-hundred-nucleotide-long transcript with a 33-base pair hairpin loop. The microprocessor complex (composed of Drosha and DGCR8) cleaves the pri-miRNA to produce a 60-nt pre-miRNA, which will be transported to the cytoplasm in an Exportin5/RanGTP-dependent manner. The RISC loading complex cleaves the pre-miRNA into a 22-nt miRNA duplex in the cytoplasm. Helicase activity unwinds the duplex into a passenger strand, which is later degraded, and a mature miRNA that is loaded into the AGO family of proteins to form a miRNA-induced silencing complex (RISC). Created with BioRender.com.

In the canonical biogenesis pathway, miRNAs are transcribed by RNA Polimerase II (RNA Pol II) as part of a hairpin structure within a larger transcript - the primary miRNA (pri-miRNA) (Bartel, 2018). Inside the nucleus, the pri-miRNA is processed to produce the precursor miRNA (pre-miRNA), which is subsequently exported to the cytoplasm and cleaved into a miRNA duplex. Helicase activity results in the unwinding of the duplex into a passenger strand and a functional mature miRNA. The first one is degraded, while the functional strand is assembled into a RISC (RNA-induced silencing complex) containing Argonaute (AGO) proteins. The RISC is guided to its target transcripts by the functional strand, which has a seed region (nucleotides 2–7) complementary to a region on the target mRNA, usually in the 3′UTR (Bartel, 2018). Figure 4 shows the biogenesis of miRNAs and the main proteins involved in the process. The generalization of massive parallel sequencing technologies, in particular methods for small RNA sequencing, has significantly simplified the systematic identification of these molecules in a wide range of systems. Notwithstanding, the detection of a small sized molecule is not proof that it is a miRNA or any other kind of functional sncRNA, as it may just represent an intermediate degradation product of a larger transcript. In order to be considered a miRNA, the detected molecules are expected to present several of key features, in particular those linked to specific aspects of the canonical biogenesis pathway. However, the restrictive application of this rationale has deterred the identification of novel, *bona fide* miRNA species that use alternative biogenesis pathways, now known to be encoded in both metazoan and viral genomes (Xie and Steitz, 2014). As such, studying viral miRNAs presents specific challenges to be addressed, compounded by the difficulties emerging from the fact they can only be found within an infected cell. As such, sample collection methods must be carefully designed to capture the presence and diversity of viral miRNAs, which may only be expressed in the context of a natural infection. However, access to such samples may be very limited and/or require working under stringent bio-safety protocols, forcing researchers to rely on more artificial, laboratory-adapted infection models. Even with high sensitivity RNA-sequencing methods, the discovery of v-miRNAs will tend to be masked by the predominance of host derived molecules or the presence of similar sequences in the host genome. Once candidate v-miRNA molecules are detected, interpreting the functional relevance and regulatory roles of these molecules requires meticulous experimental validation and functional characterization to distinguish them from natural degradation products. Therefore, advancing our understanding of viral miRNAs requires overcoming these challenges through well-designed strategies and comprehensive approaches, to reveal the intricate roles of viral miRNAs in viral replication, pathogenesis, and host immune responses.

**FIGURE 5:**
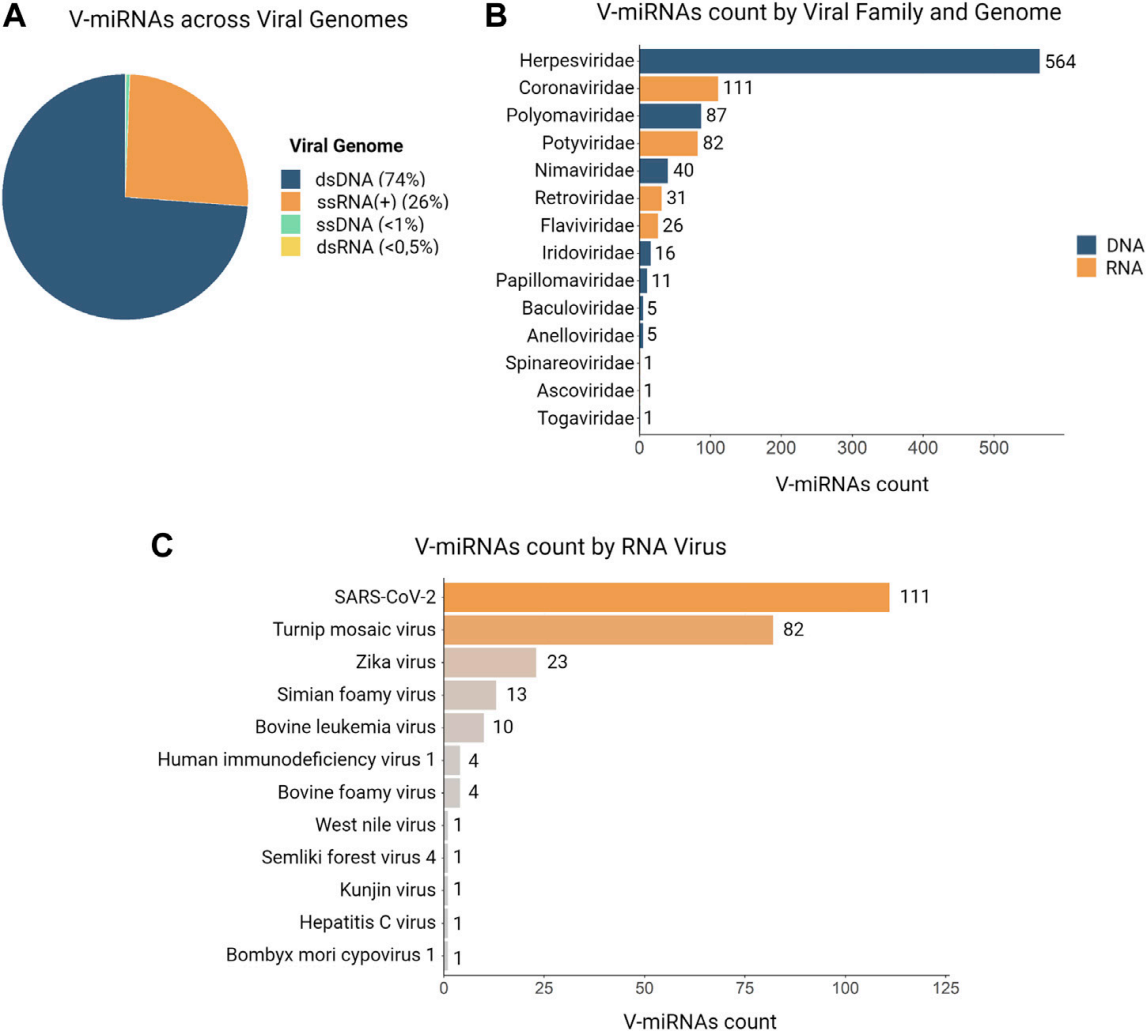
Distributions of v-miRNAs present in databases according to genome, viral family and specie. In total, we identified 981 v-miRNAs encoded by 52 viruses and distributed across 14 viral families in three different databases (VIRmiRNA, miRbase, and ViRBase). **(A)** A pie chart illustrates the abundance of v-miRNAs according to their genome, with ∼74% of entries corresponding to DNA genomes and ∼26% to RNA viruses. **(B)** The distribution of v-miRNAs entries by viral family shows that Herpesviridae has the most identified v-miRNAs, followed by Coronaviridae and Polyomaviridae, while some viral families such as Togaviridae, Ascoviridae, and Spinareoviridae have only one v-miRNA entry. **(C)** Analysis of the count of v-miRNAs entries per RNA virus reveals that SARS-CoV-2 has the largest number of identified v-miRNAs, followed by Turnip mosaic virus and Zika virus. Additionally, we found only one v-miRNA present in the databases for West Nile virus, Semliki forest virus 4, Kunjin virus, Hepatitis C virus, and *Bombyx mori* cypovirus.

The first viral-encoded miRNA (v-miRNA) was identified in 2004 in the Epstein-Barr virus (EBV) through the cloning of small RNAs in an infected cell line (Pfeffer et al., 2004). To this date, the majority of known v-miRNAs are encoded by DNA virus genomes, mainly in the Herpesviridae family (Mishra et al., 2020) (Figure 5). Given that DNA viruses rely on the host cell gene expression pathway, viral transcripts can also be processed through the canonical miRNA biogenesis pathway. This in turn makes the identification of *bona fide* v-miRNAs a much simpler process. Although the existence of miRNAs encoded by RNA viruses has been the object of some dispute, as discussed below, several studies published throughout the years provide convincing evidence for the existence of these molecules. There are currently three main miRNA databases that contain information about v-miRNAs: miRbase (Kozomara et al., 2019), VIRmiRNA (Qureshi et al., 2014), and ViRBase (Li et al., 2015) (Table 1). Together, these databases comprise approximately 1,000 entries corresponding to both experimentally validated and predicted v-miRNAs, encoded by 14 viral families. Compilation of the information provided in these databases reveals that ∼26% of all reported v-miRNAs are encoded by RNA viruses, with Coronaviridae being the family with the highest number of reported v-miRNAs (111 v-miRNAs), followed by Potyviridae (82 v-miRNAs) and Retroviridae (31 v-miRNAs) (Figure 5). These numbers highlight a huge lack of information regarding viral sncRNAs, in particular those encoded by RNA viruses, and the importance of performing more focused studies. Indeed, although SARS-CoV-2 was only identified in 2020, the considerable attention it received due to the COVID-19 pandemic led to its rank as the RNA virus with the largest number of database entries. This fact underscores the impact of the recent pandemic in bringing the study of v-miRNAs encoded by RNA viruses back into the spotlight. However, it is important to note that several miRNAs reported in the literature are not present in these databases, partly due to their lack of updating. A list of v-miRNAs encoded by RNA viruses with relevance to human health, including manually curated examples from the literature, can be found in Table 2.

**TABLE 1:** Summary of virus-encoded miRNAs across miRNA databases.

DataBase	Method	V-miRNAs	Species	Families	Last update	References	Web address
miRBase	Predicted and experimental evidence	31*/530	4*/34	1*/6	2019	Kozomara et al. (2019)	https://www.mirbase.org/
VIRmiRNA	Experimental evidence	113*/823	7*/44	4*/12	2014	Qureshi et al. (2014)	http://crdd.osdd.net/servers/virmirna/index.html
ViRBase	Predicted and experimental evidence	50*/475	6*/25	4*/9	2021	Li et al. (2015)	http://www.rna-society.org/virbase/index.html

The table provides information on databases that contain data on virus-encoded microRNAs (v-miRNAs), including the method of identification, number of v-miRNAs identified, number of species and families from which the v-miRNAs were identified, the year of the last update, reference, and web address of the database. The asterisks indicate the number of v-miRNAs encoded by RNA viruses *versus* the total number of miRNA entries in each database.

**TABLE 2:** Viral miRNAs encoded by RNA viruses.

Virus	V-miRNA	Targets	Functions	References
**Coronaviridae**				
SARS-CoV-2	Several putative miRNAs	Multiple cellular targets	Processes of viral infection and host immune response; Signaling pathways; Epigenetic regulation pathways	Saini et al. (2020), Aydemir et al. (2021), Liu et al. (2021), Roy et al. (2021)
**Potyviridae**				
Tumv	tumv-miR-s1	HVA22D in Arabidopsis	Cellular defense pathway	Ramesh et al. (2021)
	tumv-miR-s1			
**Flaviviridae**				
WNV	kun-miR-1	LOC5573053; GATA4	Promotion of viral replication	Hussain et al. (2012)
ZIKA	Several putative miRNAs	Multiple cellular targets	Immune surveillance and other biological pathways	Islam et al. (2019)
DENV	DENV-vsRNA-5	NS-1	inhibition viral replication	Hussain and Asgari (2014)
**Retroviridae**				
BFV	bfv-miR-BF2-3p/5p	ANKRD1; Bif1	Regulation of viral replication	Whisnant et al. (2014), Cao et al. (2020)
	bfv-miR-BF1-3p/5p	Unknown	Unknown	
BLV	blv-miR-b1-3p/5p	Unknown	Unknown	Kincaid et al. (2012), Rosewick et al. (2013)
	blv-miR-b2-3p/5p	Unknown	Unknown	
	blv-miR-b3-3p/5p	Unknown	Unknown	
	blv-miR-b4-3p	Mimic of host miR-29	Cell proliferation	
	blv-miR-b4-5p	Unknown	Unknown	
	blv-miR-b5-3p/5p	Unknown	Unknown	
	bfv-miR-BF1-3p/5p	Unknown	Unknown	
ALV-J	miRNA-like sequences*	E (XSR) element in the 3′UTR	Tumorigenesis	Yao et al. (2014)
HIV	hiv1-miR-H1	AATF; vpr; host miR-140	Apoptosis inhibition; Vpr expression	Kaul et al. (2009), Lamers et al. (2010)
	hiv1-miR-N367	Nef; PABPC4	Promotes viral infection	Omoto et al. (2004), Omoto and Fujii (2005)
	hiv1-miR-TAR-3p/5p	Apoptosis-related genes	Apoptosis inhibition and viral propagation	Ouellet et al. (2008)
	miR-H3*	TATA box in the 5′LTR	Promotes viral replication	Zhang et al. (2014)
SFV	sfv-mir-S1	Unknown	Unknown	Kincaid et al. (2014)
	sfv-mir-S2	Unknown	Unknown	
	sfv-mir-S3	Unknown	Unknown	
	sfv-mir-S4	Mimic of host miR-155	Cell proliferation	
	sfv-mir-S5	Unknown	Unknown	
	sfv-mir-S6	Mimic of host miR-132	Immunoevasion	
	sfv-mir-S7	Unknown	Unknown	
**Picornaviridae**				
HAV	hav-miR-N1-3p*	MAVS	Cellular antiviral pathways	Shi et al. (2014a)
	hav-miR-1-5p*	Unknown	Inhibition of viral replication	Shi et al. (2016)
	hav-miR-2-5p*			
**Filoviridae**				
EBoV	EBOV-miR-1-3p/5p*	c-MET, Activin	Cellular signaling pathways	Liang et al. (2014), Duy et al. (2018)
	EBOV-miR-T3-3p*	and KPNA1, importin-alpha5		Duy et al. (2018)
	EBOV-miR-T3-3p*			
	EBOV-miR-VP-3p*	Unknown	Possible biomarker for early stages of infection	Chen et al. (2016)
**Orthomyxoviridae**				
IAV	miR-HA-3p*	PCBP2	Regulates cytokine production and viral infection	Li et al. (2018)

Examples of v-miRNAs and their predicted targets and functions, across different viral families. The table includes information on v-miRNAs present and not present (*) in miRNA databases and their associated cellular processes, including viral replication, immune response, and tumorigenesis. SARS-CoV-2, Severe acute respiratory syndrome coronavirus 2; Tumv, Turnip mosaic virus; WNV, West Nile virus; DENV, Dengue virus; BFV, Bovine foamy virus; BLV, Bovine leukemia virus; ALV-J, Avian leukosis virus J strain; HIV-1, Human immunodeficiency virus-1; SFV, Simian foamy virus; HAV, Hepatitis A virus; EBOV, Ebola virus; IAV, Influenza A virus.

We next present a detailed review of sncRNAs encoded by different families of RNA viruses. We focus mainly on miRNAs, regarding which the available information is more detailed, but also go through other more recent and/or less recognized classes of v-sncRNAs. As previously mentioned, until recently it was generally assumed that RNA viruses did not encode miRNAs. Several arguments have been used as a justification for this, with two key points standing out (Cullen, 2010; Aguado and tenOever, 2018). First, since most RNA viruses replicate in the cytoplasm, it was assumed that viral RNAs could not interact with the nuclear microprocessor complex and thus enable the miRNAs biosynthesis. Second, the production of canonical miRNAs from a pri-miRNA hairpin was suggested to result in unproductive cleavage of the viral genome and its transcripts (Houzet and Jeang, 2011; Harwig et al., 2014). However, viruses can hijack cellular mechanisms to the benefit of their replication efficiency and have evolved alternative pathways for their biogenesis processes. In the last decade, this has been abundantly shown by several studies, which have come to contradict the old adage that RNA viruses do not encode miRNAs (see Withers et al., 2019).

## 2 Viral sncRNAs in the virus-host strike-counterstrike interplay

As parasites, all viruses complete their life cycle within host cells by taking advantage of their translational machinery. Among RNA genome viruses, retroviruses further rely on the host transcriptional machinery to be able to synthesize both their genome and mRNAs. In parallel, following viral infection, host cells activate antiviral mechanisms to halt the viral offensive. This causes viruses to adapt quickly to avoid host cell barrages, a process known as innate immune evasion (Beachboard and Horner, 2016). To guarantee their survival, RNA viruses interact with a great number of host cellular components. This is achieved by encoding viral proteins that inhibit, interfere with, or promote host gene expression (Nelemans and Kikkert, 2019). In addition, due to their generally small genome, viruses have a limited coding potential (Withers et al., 2019). Therefore, the use of small non-coding RNAs (sncRNAs), both viral and derived from the host, to regulate host-virus interactions may confer advantageous means to control host cell functions (Withers et al., 2019).

**FIGURE 6:**
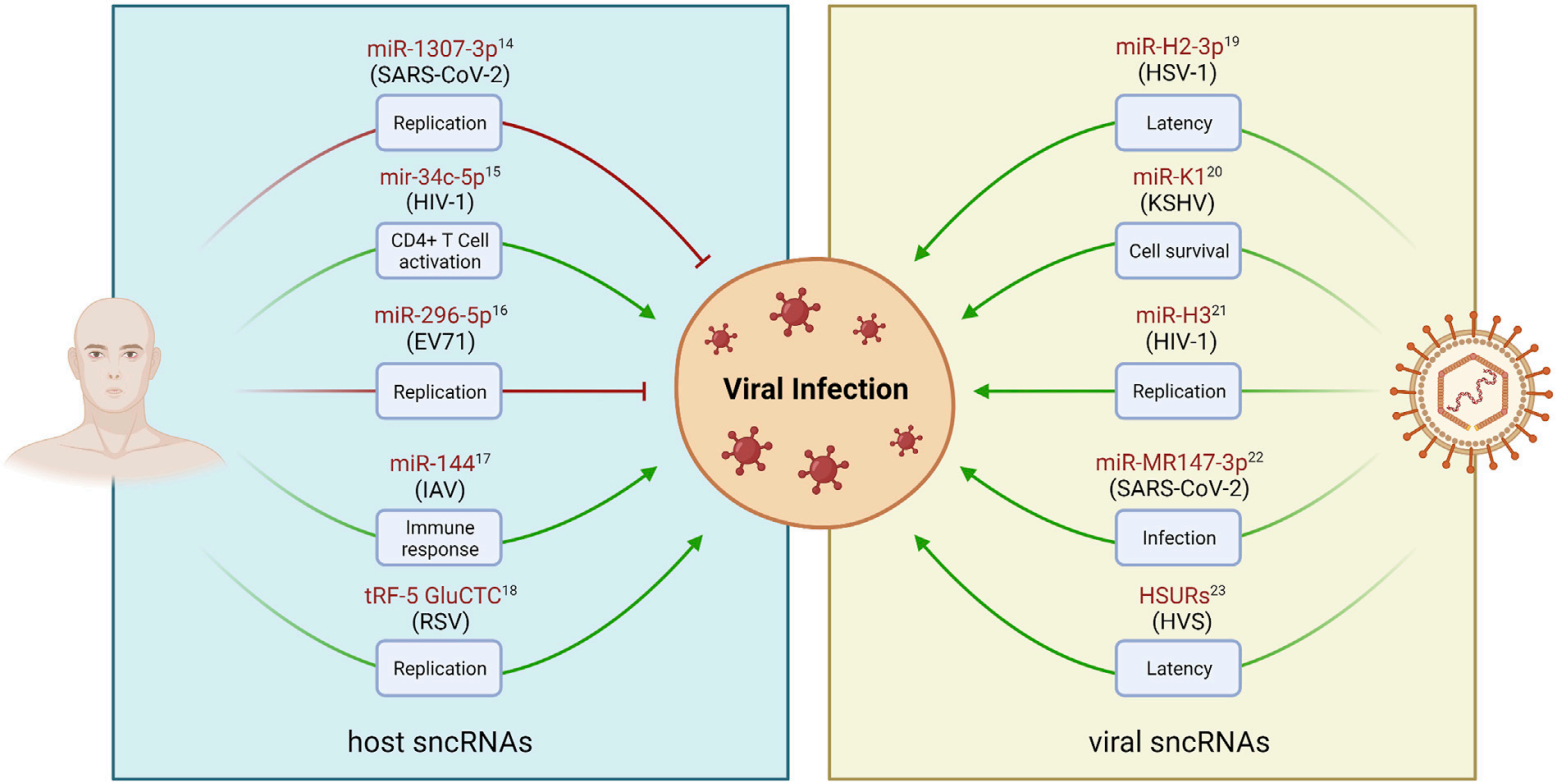
Examples of sncRNAs-mediated regulation of viral infections. Viral and host derived sncRNAs play crucial roles in regulating various stages of viral infection and pathogenesis, such as replication, cell activation, latency, and cell survival. Host sncRNAs can be regulated in response to viral infection (left panel). This can lead to the inhibition of replication as an antiviral mechanism (red arrows). Conversely, virus-dependent regulation of sncRNA levels may result in the enhancement of viral replication (green arrows). DNA and RNA viruses have also been found to encode sncRNAs, such as miRNAs, that can modulate both viral and host genes to facilitate viral infections (right panel). sncRNA names are represented in red, and the associated virus modulating (left panel) or encoding (right panel) their expression is shown in parenthesis. Created with BioRender.com.

The majority of viral ncRNAs have been identified in DNA viruses, mostly associated with the Herpesviridae family. However, recent advances in high-throughput sequencing have revealed that many other viruses express ncRNAs for their benefit, including RNA viruses (Withers et al., 2019). Notwithstanding, the fact that RNA genomes can encode miRNAs was only more recently accepted. In the last decade, v-sncRNAs earned a growing interest due to their function as potentially powerful regulators of host and viral gene expression, and their interactions with several key host pathways (Tycowski et al., 2015; Withers et al., 2019). These studies highlighted the importance of characterizing these molecules to achieve a deeper global understanding of the viral mechanisms of infection. Conversely, host sncRNAs have also been shown to interact with viruses, either to promote their replication, latency, and survival, or to inhibit viral genome elements as a mechanism of antiviral response (Girardi et al., 2018). Therefore, the study of virus-host interactions is critical for understanding the viral life cycle and the control of viral infections. Examples of sncRNA-mediated regulation of viral infection by both viral and host molecules are widely present in the literature (Umbach et al., 2008; Cazalla et al., 2010; Gottwein and Cullen, 2010; Zheng et al., 2013; Zhang et al., 2014; Deng et al., 2015; Amaral et al., 2017; Rosenberger et al., 2017; Balmeh et al., 2020; Liu et al., 2021) are shown in Figure 6.

### 2.1 ssRNA (+) virus encoded miRNAs

#### 2.1.1 Flaviviruses

The first demonstration of the ability of a single stranded RNA virus genome to encode microRNAs comes from the study of the West Nile virus (WNV), a member of the Flaviviridae family (Hussain et al., 2012). Flaviviruses include more than 130 species (Hatcher et al., 2017), including several human pathogens (Pierson and Diamond, 2020). In addition to WNV, two other flavivirus species have been proposed to be capable of producing v-miRNAs: the Dengue (DENV) and Zika (ZIKA) viruses. All three viruses are mosquito-borne, and they can cause mild to serious diseases that have a significant impact on human health (Wilder-Smith et al., 2017).

The flavivirus genome contains 5′ and 3′ untranslated regions containing multiple stem loops (SL) that protect the viral genome from degradation. The 3′ end region terminal SL has been shown to perform several critical functions related to host-viral interactions (Yu et al., 2008). Additionally, this region leads to the production of a 0.3–0.5 Kb non-coding RNA molecule, the subgenomic flavivirus RNA (sfRNA), which is essential for viral pathogenicity (Pijlman et al., 2008; see below). A bioinformatic search for potential miRNA precursors in the 3′SL led to the identification of a small miRNA-like molecule, KUN-miR-1, as the first miRNA encoded by a cytoplasmic RNA virus (Hussain et al., 2012). In mosquito cell lines, this v-miRNA was shown to be produced by a Dicer-dependent mechanism and to be able to inhibit the expression of sensor constructs and facilitate viral replication. This effect was shown to occur through the upregulation of the transcriptional activator GATA4 mRNA, a direct target of KUN-miR-1. Although non-canonical, multiple studies have demonstrated that miRNAs can act as positive regulators of their targets (Vasudevan et al., 2007). Unexpectedly, KUN-miR-1 does not seem to be expressed in human cells infected with WNV. This difference was suggested by the authors to be due to an additional regulatory element in mammalian cells that prevents the conversion of the SL RNA into a mature v-miRNA (Hussain et al., 2012). In contrast, in a later study on DENV encoded v-miRNAs, DENV-vsRNA-5 was identified in both mosquito and human DENV-2-infected cells (Hussain and Asgari, 2014). DENV-vsRNA-5 was proposed to negatively regulate viral replication by targeting the non-structural protein-1 (NS1) coding region in the viral genome, suggesting that v-miRNAs can also contribute to attenuating viral replication (Hussain and Asgari, 2014). Notwithstanding, the functional evidence presented in this study was subjected to significant criticism, raising doubt over the biological relevance of the reported sequences (Skalsky et al., 2014).

The Zika virus is an emerging Arbovirus of the Flaviviridae family closely related to Dengue and West Nile, which exhibits uncommon health consequences for humans and is responsible for several outbreaks around the globe. The most recent outbreak was reported in Brazil in May 2015, spreading across other continents and resulting in an increased number of congenital malformations such as microcephaly, leading to a spur in research interest on the molecular mechanisms of viral replication (Islam et al., 2019). Using bioinformatic approaches, a recent study identified 12 candidate viral pre-miRNAs and 47 mature miRNAs encoded in the Zika genome, proposing that these molecules modulate host genes involved in immune signaling and nervous system functions (Islam et al., 2019). While this evidence represents preliminary results that must be confirmed experimentally, these putative v-miRNAs are already present in miRNA databases, justifying their reference here.

#### 2.1.2 Coronaviruses

Coronaviruses (CoV) are a family of pleomorphic, enveloped single-stranded RNA viruses with a diameter ranging from 80 nm to 120 nm, that was identified in the 60s as causing common colds (Tyrrell and Bynoe, 1966). It was only recently that severe acute respiratory syndromes (SARS) caused by coronaviruses emerged, with the SARS-CoV virus being responsible for the 2003 SARS epidemic (Perlman and Netland, 2009), and the SARS-CoV-2 virus for the recent COVID-19 pandemic (Hu et al., 2021). As a result of the highly focused, global research effort to understand its genome and replication cycle, multiple published studies describe the existence of SARS-CoV-2 encoded v-miRNAs. The first research to demonstrate the presence of v-miRNAs in coronavirus genomes dates to a study of SARS-CoV in 2017 (Morales et al., 2017). However, it was during the recent COVID-19 pandemic that the extensive reporting of SARS-CoV-2 encoded miRNAs through computational approaches opened the door to the unprecedented identification of a large number of candidate miRNAs encoded by RNA viruses. A first genome-wide computational prediction identified 26 candidate mature v-miRNAs from the SARS-CoV-2 genome targeting host cell apoptotic processes and signaling pathways (Saini et al., 2020). Later, another computational work predicted 40 SARS-CoV-2 putative miRNAs able to target several host genes and regulate NFKB, JAK/STAT, and TGFB signaling pathways, as well as cellular epigenetic regulation pathways (Aydemir et al., 2021).

It is important to recognize that such bioinformatic approaches are bringing up important discussions about miRNAs encoded by the SARS-CoV-2 virus. However, caution must be taken because any computational prediction of candidate miRNAs and their targets requires experimental validation. In this regard, several experimental studies have been conducted to identify strong v-miRNA candidates and understand their role during viral infection. For example, experimental validation suggests that SARS-CoV-2 v-miR-147-3 is associated with infection stages in the Vero E6 cell model (Liu et al., 2021). Overexpression of this miRNA reduced expression of the human targets EXOC7, RAD9A, and TFE3, which are involved in membrane addition, polarized exocytosis, signaling of transforming growth factor-beta, and regulation of fat and glucose metabolism (Liu et al., 2021). Moreover, an independent study showed that v-miR-147-3 enhances the expression of TMPRSS2 in the gut and increases the capacity of the virus for transmission (Liu et al., 2020). Other SARS-CoV-2 v-miRNAs have been identified in Vero E6 and Calu-3 infected cells (v-miRNA-N-28612, v-miRNA-N-29094, and v-miRNA-N-29443) (Meng et al., 2021). These miRNAs are encoded in the nucleocapsid gene (N), which critically regulates pro-inflammatory cytokines and lung pathology (Meng et al., 2021). A Northern blot assay-based study confirmed that SARS-CoV-2 encodes the miRNA-like vmiR-5p in its ORF7a gene, which may also be involved in the pathogenesis of the virus (Pawlica et al., 2021).

Over the last year, the list of v-miRNAs proposed to be encoded by different regions of the SARS-CoV-2 genome has grown exponentially and a review focused only on this topic would be necessary for a more global view of the role of these v-miRNAs in virus-host interaction. As a result of the recent interest in studying Coronavirus-encoded sncRNAs, the Coronavirus family is now the second family with the greatest number of v-miRNAs described in miRNA databases (111 v-miRNAs), only behind the dsDNA Herpesviridae family. Further, SARS-CoV-2 has more putative v-miRNAs described than any other RNA virus (Figure 5). In summary, these v-miRNAs have been suggested to regulate several human targets and pathways including apoptosis, regulation of cytoskeleton dynamic, viral replication, viral exocytosis and release, signaling, and much more, suggesting potential relevance in the development of successful therapeutics (reviewed in (Bhattacharyya and Biswas, 2020; Omer and Kubra, 2021; Paul et al., 2022)).

#### 2.1.3 Potyvirus

Potyviruses (members of Potyviridae family) are the largest group of plant-infecting viruses and include the most economically relevant crop pathogens, thus being the most well studied group of plant virus (Yang et al., 2021). This fact probably justifies that this family of RNA viruses is the second with more v-miRNAs listed in the VIRmiRNA database (Figure 5; Table 2), all encoded by the Turnip mosaic virus (TuMVmv). Unlike other entries in the database, these v-miRs are referenced with a patent submission number and to the best of our knowledge, they have been identified as part of an unpiblished doctoral thesis and are not further cited or described in the scientific literature (Ramesh et al., 2021). Thus, there is very limited information regarding the functional validation of these sequences. According to a review by (Ramesh et al., 2021), two of the TuMV-encoded v-miRNAs (TuMV-mir-S1 and TuMV-mir-S2) were subjected to experimental validation and shown to downregulate the host stress-responsive gene HVA22D in Arabidopsis, which is crucial for the cellular defense pathway and regulated by abscisic acid. The same review mentions several other miRNAs encoded by this family of plant viruses. Due to this and the fact that they are not present in miRNA databases, they will not be further discussed here. However, given the prevalence of TuMV-encoded miRNAs in the databases we reviewed, mentioning them in this section was mandatory. Just like the case of SARS-CoV-2 miRs, this specific example underscores how the number of known RNA virus encoded v-miRs may be influenced by the lack focused research efforts.

#### 2.1.4 Picornaviruses

The Picornaviridae family, encompassing several groups of picornaviruses, is an archetypal family of non-enveloped (+) ssRNA viruses, many of which are relevant human and livestock pathogens, including the agents causing polyomielitis, the common cold, hepatitis A and foot-and-mouth disease (Tuthill et al., 2010). The replication of ssRNA viruses involves the transcription of an antigenome RNA by a viral RNA-dependent RNA Polymerase (RdRP), which then uses this molecule as a template for the synthesis of the new viral genomes (Rampersad and Tennant, 2018). The presence of v-miRs in both the HAV genome and anti-genome was assessed by small RNA-seq characterization of HAV infected human fibroblasts (Shi et al., 2014b). This led to the identification of several v-sncRNA molecules that accumulate specifically in infected cells, three of which were experimentally shown to display hallmark features of miRNAs. The genome-derived hav-miR-1-5p and hav-miR-2-5p, and the antigenome-derived hav-miR-N1-3p were shown to be encoded in a precursor hairpin sequence that undergoes Dicer-dependent processing, and to be able to regulate the expression of a reporter vector containing complementary sequence elements in the 3′UTR (Shi et al., 2014a; Shi et al., 2014b). Further studies by the same authors suggest that these v-miRs may primarily target the HAV genome and anti-genome sequences to reduce viral RNA abundance, eventually supporting the establishment of persistent infection states (Shi et al., 2016). A similar mechanism had been previously observed in DENV-infected cells (Hussain and Asgari, 2014). Notwithstanding, the biological roles of these HAV-derived miRNAs in regulating host–virus interactions require further investigation.

### 2.2 ssRNA (−) virus encoded miRNAs

#### 2.2.1 Filoviruses

The family Filoviridae derives its name from the frequent filamentous shape presented by its member species, characterized by a single-stranded, non-segmented, antisense RNA genome (Kuhn et al., 2019b). Several of the group members are highly virulent human pathogens, causing hemorrhagic fevers, namely, the Marburg (MARV) and Ebola virus (EBOV). The recent outbreaks of infections by EBOV in Western Africa have resulted in an unprecedented number of study cases supporting a major revision of filovirus disease classification by the World Health Organization, as well as bolstering research efforts on this virus (Kuhn et al., 2019a). To date, several mature v-miRNAs and pre-miRNAs have been proposed to be encoded by the Ebola virus. However, none of these miRNAs are reported in miRNA databases (Table 2). The first study proposing the ability of EBOV to encode miRNAs used bioinformatic analysis to predict that three mature miRNAs (EBOV-miR-1-5p/-3p, and miR-2-3p) were formed from two pre-miRNAs (EBOV-pre-miR-1 and EBOV-pre-miR-2) as a result of Dicer-dependent processing (Liang et al., 2014). In the following years, several other candidate v-miRNAs were identified as a result of genome-wide screening and experimental validation (Teng et al., 2015; Chen et al., 2016; Liu et al., 2016; Prasad et al., 2020). The EBOV-miR-1-5p analog of human miR-155 was proposed to inhibit importin-alpha5 in HEK293T, leading to immune evasion in EBOV-infected cells (Liu et al., 2016). In addition, EBOV-miR-VP-3p, derived from the viral VP40-coding region, was shown to be abundantly expressed in the sera of EBOV-infected individuals at early stages of infection, suggesting it may serve as a biomarker for early detection of EBOV infection (Chen et al., 2016). More recent research confirmed the accumulation of sncRNAs in bat and human cells infected by EBOV and MERV, including sequences very similar to previously reported EBOV-encoded v-miRNAs (Prasad et al., 2020). However, in spite of a rather significant effort to demonstrate the functionality of EBOV-derived molecules in the miRNA pathway, the authors failed to detect any silencing activity on reporter constructs or a measurable effect on virus replication (Prasad et al., 2020). Thus, although there is evidence for controlled production of viral sncRNAs in EBOV and MARV infected cells, these results raise significant questions regarding their putative role as v-miRs.

#### 2.2.2 Orthomyxoviruses (influenza viruses)

The Orthomyxoviridae virus family of enveloped, negative-strand, segmented RNA viruses include five genera, of which Influenza A viruses (IAVs), which exist as populations of quasi-species, are the only one that poses significant risk of zoonotic potential, host switch, and recurrent generation of pandemic strains (Taubenberger and Kash, 2010). Among RNA viruses, IAVs are unusual in that they replicate and transcribe in the host cell nucleus, therefore altering the rules regarding the ability to interact with the canonical host miRNA machinery. Among this group, the H5N1 IAV is a responsible for the highest mortality among influenza viruses (Korteweg and Gu, 2008) and is the only Orthomyxovirus reported to encode miRNAs. In spite of a first small RNA sequencing study of cells infected with the H3N2 IAV failing to identify any v-miRs, while reporting the existence of high abundance leader sncRNAs (leRNAs; see below; Umbach et al., 2010), a later study focusing on H5N1-infected cells came to different results (Li et al., 2018). The most abundant sncRNA detected in these cells was shown to originate from a stem-loop precursor cleaved by Ago2 and to display key characteristics of v-miRs, thus being named miR-HA-3p (Li et al., 2018). Of note, his v-miRNA is not included in any miRNA database. However, in addition to its expression being validated using multiple methods and in different host cell lines, its function has also been addressed experimentally. miR-HA-3p has been shown to post-transcriptionally inhibit the production of PCBP2, one of the proteins involved in cytokine signaling and host antiviral response (Li et al., 2018). By blocking PCBP2 activity, miR-HA-3p suppresses inflammatory cytokine production and enhances viral infection, providing evidence it may act as a bonafide v-miR. Notwithstanding, given the similarities between different IAV quasi-species, the discrepancies between the studies of H3N2 and H5N1 are intriguing and warrant further investigation.

### 2.3 Retrovirus encoded miRNAs

Retroviruses are unique among viruses with RNA genomes because they encode a reverse transcriptase that generates a cDNA copy, which is then integrated into the host genome. This pro-viral DNA is transcribed by the host transcriptional machinery to produce both full length RNA genomes and mRNAs encoding viral proteins (Coffin et al., 2021). The Retroviridae are split into two subfamilies separating the “canonical” retroviruses (Orthoretroviridae), which contain viruses with pandemic potential and causing multiple pathologies, including the human immunodeficiency viruses HIV-1 and 2, and the human tumor leukemia virus (HTLV1); and the foamy viruses (Spumaretroviridae), apathogenic and widespread in primate species populations and present in a broad range of other mammals, but so far only found in humans as a consequence of zoonotic infection (Meiering and Linial, 2001).

Computational analyses of retroviral genomes have supported the initial identification of putative retrovirus-encoded miRNAs by several independent groups (Harwig et al., 2014), (Table 2). The Bovine Leukemia Virus (BLV) is one of the five RNA viruses with the highest number of miRNA sequences reported to date (10 v-miRNAs on databases; Figure 5; Table 2), of which BLV-miR-B4-3p was the first to be functionally validated. BLV-miR-B4-3p was shown to be produced via a Drosha-independent, non-canonical miRNA-biogenesis pathway (Kincaid et al., 2012). Unlike canonical miRNAs, its precursor is transcribed by RNA polymerase III (Pol III) instead of RNA polymerase II, producing a subgenomic transcript of approximately 70 nucleotides that can form a stem-loop hairpin. This harpin is exported to the cytoplasm and subsequently processed by Dicer (Kincaid et al., 2012). BLV-miR-B4-3p was shown to mimic the seed sequence of host miR-20, a miRNA associated with B cells tumorigenesis in mice, which may indicate the association of this miRNAs with cell proliferation of infected cells and BLV-induced tumorigenesis (Kincaid et al., 2012). The Pol III-dependent synthesis of BLV-miR-B4-3p, as well as of the other eight v-miR candidates predicted in this study was validated in the context of natural infections by deep-sequencing of a BLV ovine model of leukemia/lymphoma, which led to the identification of two additional v-miR candidates (Rosewick et al., 2013). Strikingly, v-miRs were found to accumulate to levels corresponding to 40% of all detected miRs, both in experimental and natural malignancy, and confirmed to associate to Ago2. A more recent study has expanded these observations to provide substantial evidence that BLV v-miRs are functional genetic elements that regulate viral replication and contribute to oncogenesis (Gillet et al., 2016). Collectively, these studies unequivocally establish that retroviruses are able to encode functional v-miRs. Of note, the RNA-seq study by Rosewick and others was not able to detect any candidate v-miRs expressed in HTLV-1 infected cells, in line with other reports on the HIV-1 retrovirus.

The ability of HIV-1 to produce miRNAs is probably the most controversial debate within the retrovirus family. Like other miRNAs, the earliest studies regarding HIV-encoded miRNAs relied on computation approaches. One of the first works on the topic demonstrated that all the five regions of the HIV-1 genome (RNA—trans-activation response (TAR) element, Gag-CA, Gag-Pol frameshift, Nef, and 3′LTR) contain stem-loop structures that are potential miRNA-yielding hotspots (Bennasser et al., 2004). The first putative miRNA encoded by the HIV-1 genome to be described and experimentally validated was miR-N367 (Omoto et al., 2004). This molecule was detected in T cells persistently infected with HIV-1 IIB and SF2 strains and is derived from a 70-nucleotides-long structure in the Nef/LTR overlapping region. miR-N367 has been shown to negatively target the response elements of the LTR U3 region inhibiting the LTR’s promoter activity (Omoto et al., 2004; Omoto and Fujii, 2005). However, this candidate miRNA was not found in later studies that screened infected HeLa cells and chronically infected T cells using HIV-1 LAV strains (Pfeffer et al., 2005; Lin and Cullen, 2007). Another reported HIV-encoded miRNA is miR-H1. This v-miRNA is located downstream of two NF-kB-biding sites in the viral LTR and downregulates the apoptosis-antagonizing transcription factor (AATF) (Kaul et al., 2009; Lamers et al., 2010). Furthermore, two independent studies identified TAT-derived miRNAs. Ouellet et al. additionally reported miR-TAR-5p and miR-TAR-3p, produced as a result of asymmetrical processing of the HIV-1 TAR element (Ouellet et al., 2008). These miRNAs were shown to target host genes related to apoptosis, cell survival, and viral replication. More recently, deep sequencing analysis of AGO-1 bound HIV-1 RNAs demonstrated that the 3′ region of the TAR hairpin is processed into a miRNA-like molecule (Harwig et al., 2016). The RT region of the HIV-1 has also been associated with the production of a v-miRNA, named miR-H3, which targets the TATA box in the viral 5′LTR promoter and upregulates viral RNA transcription and protein synthesis (Zhang et al., 2014).

Next-generation sequencing technologies (NGS) have been used in an attempt to validate the reported HIV-1 miRNAs, with contradictory results reported. A study that relied on sequencing by oligonucleotide ligation and detection (SOLiD) technology-based identified 5 v-miRNA candidates, including the previously described miR-TAR (Schopman et al., 2012). In contrast, Whisnant and co-workers generated high depth Illumina small-RNA-seq libraries from several cell lines and primary human CD4^+^ T cells infected with different HIV-1 strains and did not detect the previously described HIV-encoded miRNAs or other novel candidate miRNAs (Whisnant et al., 2013). The authors concluded that the detected likely represent degradation products, and that HIV-1 does not encode microRNAs (Whisnant et al., 2013). Furthermore, a second study also reached the same conclusions when they failed to detect the incorporation of HIV-1-derived sncRNAs into the AGO2-RISC complex, despite the presence of a multitude of sncRNAs of viral origin in HIV-1 infected cells. This suggests that even if HIV-1 can produce functional sncRNAs, they may not be involved in the canonical miRNA pathway (Vongrad et al., 2015). This set of studies has fueled a strong controversy about the ability of HIV-1 to encode functional v-miRs, with a significant emphasis being placed on the significance of detecting low abundance molecules, notwithstanding their functional validation based on over-expression and the use of synthetic target reporters.

Amid this discussion, another member of the Orthoretroviridae sub-family, the Avian Leucosis Virus subgroup J (ALV-J), has been proposed to encode a miRNA produced via the canonical pathway (Chen et al., 2007; Yao et al., 2014). ALV-J miRNA seems to be encoded in the exogenous virus-specific region E element, or XSR, which is responsible for the ALV-induced tumorigenesis (Yao et al., 2014). Finally, two members of the foamy virus family have been shown to encode *bona fide* v-miRNAs (Figure 5; Table 2). Starting from the deep-sequencing of Bovine foamy virus (BFV) infected cells, Whisnant and co-workers reported the detection of three high-abundance candidate v-miRNAs and validate their ability to inhibit a reporter construct, in contrast with their previous studies of HIV-1 infected cells (Whisnant et al., 2014). These v-miRs are generated from two precursor hairpins transcribed by RNA pol III, similar to BLV encoded v-miRs. Similar findings were made for Simian foamy virus (SFV) (Kincaid et al., 2014; Hashimoto-Gotoh et al., 2020). Follow-up studies of BFV v-miRNAs revealed a critical role in viral replication (Cao et al., 2020). Of note, BFV and SFV genomes encode miRNAs in cassetes with closely spaced pri-miRNAs presenting peculiar dumbbell shaped stem-loop structures, unlike BLV miR loci, which consists of individual stem-loop structures (Materniak-Kornas et al., 2019). The striking conservation of these cassettes across different foamy virus species supports the functional relevance of the encoded v-miRs.

## 3 v-miRNAs functions and mechanisms: from enhanced immune evasion to viral replication

**FIGURE 7:**
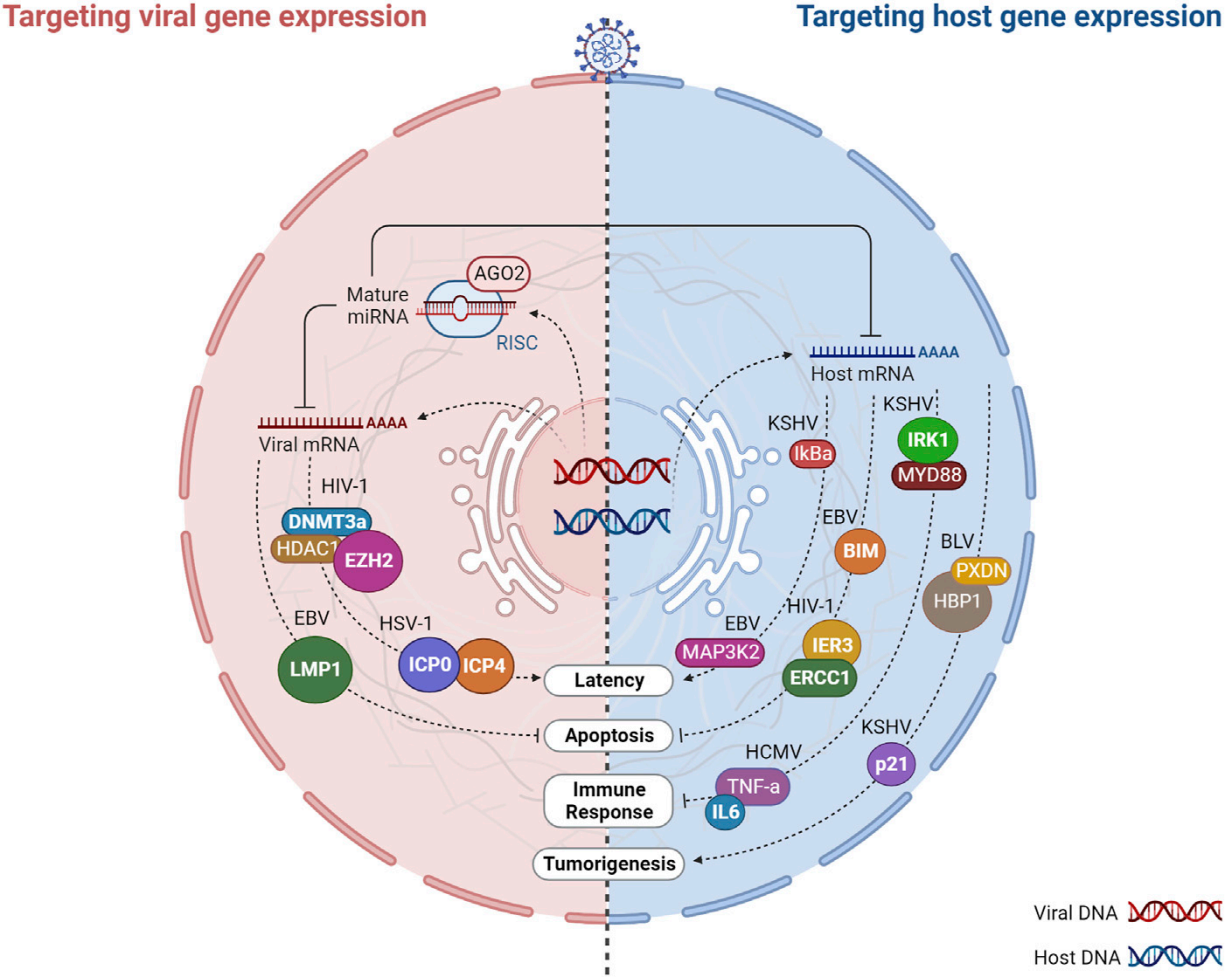
Virus-encoded miRNAs target host and viral gene expression. Virally encoded miRNAs can impact viral infection directly by regulating viral genes or indirectly by targeting host genes that play vital roles in infection. Host cells are infected by DNA or RNA viruses that replicate and produce miRNAs. After processing, viral miRNAs are incorporated into the miRNA-induced silencing complex (RISC) and target multiple arms of the immune system by regulating the expression of both host and viral mRNAs involved in viral latency, apoptosis, immune response, and tumorigenesis. The regulation of viral genes by v-miRNAs is normally associated with a mechanism that regulates the balance between the latent phase and the replication phase of the virus. In turn, the targeting of host genes allows the virus to evade anti-viral defenses and prolong the life cycle of the virus through the regulation of different biological processes such as the immune response, apoptosis, and tumorigenesis. Created with BioRender.com.

An understanding of the mechanisms of action of v-miRNAs is essential for recognizing their role in the context of the complex virus-host interactions. In light of the v-miRNA targets discovered so far, these molecules have been implicated in the regulation of immune evasion, latency, and apoptosis of host cells using two main mechanisms: regulating viral gene expression or targeting host cellular processes (Figure 7).

### 3.1 Targeting viral RNA: v-miRNAs as regulators of latent and persistent infection

The predominant reason why v-miRNAs regulate viral transcripts appears to be associated with the regulation of latency. As obligate intracellular infectious agents, viruses require continuous transmission to new susceptible individuals to be maintained in a population. Alternatively, they may develop strategies that allow their silent permanence within a host, in a state of cryptic infection, with genomic persistence and minimal gene expression, which can be reversed many years later with the production of infectious viral particles (Speck and Ganem, 2010). DNA viruses, in particular the Herpesviridae family, are the masters at taking advantage of this latent state, evading the host-immune surveillance. A virus in latency expresses only a limited number of genes, therefore it requires a promoter agent in order to restart the replication of its entire genome. v-miRNAs have the property of being non-immunogenic, making them an ideal tool for promoting the switch between latency and lytic replication through negative regulation of viral transcripts (Pfeffer et al., 2004; Speck and Ganem, 2010). Initially proposed when the first herpesvirus miRNA was discovered, this mechanism has since been described as a conserved process of this viral family (Pfeffer et al., 2004; Speck and Ganem, 2010). For example, as a result of HSV-1 encoding miR-H2-3p and miR-H6, the latency of the virus is promoted by targeting the viral reactivation factors ICP0 and ICP4, which are essential to promote viral reactivation following latency (Umbach et al., 2008). Similarly, HSV-2 latency is driven by miR-I, miR-II, and miR-III, which downregulate ICP34.5 expression, a key neurovirulence factor (Tang et al., 2009). The human cytomegalovirus (HCMV) also employs a v-miRNA-dependent strategy, which encodes miR-UL112-1. The 3′UTR of, IE1 (immediate early viral protein) is targeted by this v-miRNA, thereby promoting latency of HCMV (Grey et al., 2007). Interestingly, miRNAs encoded by the Kaposi’s sarcoma herpesvirus (KSHV) can both promote latency and control lytic reactivation. KSHV latently-infected cells were found to be less capable of reactivating lytic replication after inhibition of v-miRNAs (Ziegelbauer et al., 2009). On the other hand, according to two independent studies, KSHV mutants lacking miRNA genes exhibit increased lytic reactivation-related genes, reduced repressive marks on histones, and reduced DNA methylation, suggesting that epigenetic mechanisms may regulate latency (Lei et al., 2010; Lu et al., 2010). KSHV miRNAs such as miR-K9, miR-K1, and miR-K5 were further associated with this mechanism of immune evasion (Lei et al., 2010; Lu et al., 2010; Abend et al., 2012). In addition to these examples, other DNA viruses have been shown to promote latency or lytic reactivation (e.g., EBV, SV40). Finally, v-miRNAs encoded by DNA viruses have also been shown to target viral transcripts to reduce the activation of apoptotic pathways and hence sustain cellular viability. The latent membrane protein 1 (LMP1) is an EBV transforming agent that stimulates cell proliferation and survival via the activation of nuclear factor-kappa B (NF-kB). Although LMP1 is essential for EBV immortalization, its overexpression can significantly induce apoptosis and suppress NF-kB. EBV-miRNAs (miR-BART16, miR-BART17-5p, and miR-BART1-5p) downregulate LMP1 expression by targeting it, thereby diminishing the proapoptotic impact of LMP1 and its inhibitory effect on NF-kB (Lo et al., 2007).

Among RNA genome viruses, retroviruses are also known establish canonical latent infections, a process that has been proposed to involve RNA-dependent regulation. Indeed, an HIV-1 antisense long non-coding RNA has been proposed to assist in the recruitment of a chromatin remodeling complex composed of EZH2, the DNMT3a DNA methyltransferase, and the HDAC1 histone deacetylase, to promote transcriptional shut-down via epigenetic mechanisms (Saayman et al., 2014). The current understanding of the mechanisms behind HIV-1 latency have evolved significantly in the past few years (Pasternak and Berkhout, 2023). In addition to the original model of “deep latency” linked to transcriptional silencing, new evidence suggests that persistent infections are also maintained in cells were the virus remains active. This may involve two critical processes: limitation of the levels of RNA synthesis, namely, through the regulation of RNA metabolism; and immune evasion, through the modulation of host genes (Pasternak and Berkhout, 2023). The formation of abortive transcrips products from non-processive Tat-dependent transcription has been proposed to play a role in the process. Despite all the questions that remain regarding the existence of HIV-1 encoded v-miRs, it is noteworthy that studies addressing their biogenesis and functional validation link identified molecules to unprocessive TAR transcription (Harwig et al., 2016) and downregulation of viral (Coiras et al., 2009). Interestingly, the putative involvement of v-miRs in these processes implies that they are not expected to accumulate to high levels in actively replicating cells, offering an explanation for the reported results from deep-sequencing studies.

Although RNA viruses are typically associated to acute infections followed by immune clearance, growing evidence from the past few years has begun to reveal a different scenario of long-term persistence of viral RNA molecules in the infected host (Griffin, 2022). These processes seem to be linked to noncytolytic control of viral infection and immune evasion and may have an impact on the establishment of chronic disease conditions. Although it has been hypothesized that the detection of persistent viral RNA is due to the presence of non-infectious, viral genomic remnants that come from cells surviving the original infection, the description of cases of sexual transmission of MARV, EBOV and Zika virus months to years after recovery from acute disease suggests that RNA viruses may also resort to strategies that promote the establishment of persistent infectious states (Griffin, 2022). The fact that many v-miRs identified in RNA viruses have been proposed to downregulate viral RNA molecules fits well into a narrative of reducing pathogenicity to achieve persistence. In this regard, it is note-worthy that although most RNA virus frequently lyse cells *in vitro*, primary cells and cells infected *in vivo* are often resistant to cell death (Randall and Griffin, 2017). These examples highlight how much we still have to learn about the interactions between RNA virus and hos cells, and the role that v-miRNAs may play in modulating them.

### 3.2 Targeting host gene expression to promote viral processes

Several v-miRNAs have been shown to target host genes involved in various cellular processes critical for viral replication, including apoptosis, cell cycle regulation, and immune response. Among the conserved mechanisms attributed to v-miRNAs, the regulation of apoptotic processes and tumorigenesis stands out. By inhibiting apoptotic processes or inducing the transformation of healthy cells into tumor cells, v-miRNAs can increase the lifespan of the infected cells, thereby extending the duration of the infection within the host organism. For example, the KSHV encoded v-miR-K1 targets the cellular cyclin-dependent kinase inhibitor p21, a protein with known tumor suppressor functions, suggesting a role of this v-miRNAs in KSHV induced tumorigenesis (Gottwein and Cullen, 2010). Moreover, The Kaposi’s sarcoma-associated herpesvirus (KSHV) miRNAs, namely, miR-K12-1, miR-K12-3, and miR-K12-4-3p, have been identified as negative regulators of the apoptotic protein Casp3, promoting the inhibition of apoptotic processes (Suffert et al., 2011). Epstein-Barr Virus (EBV) BART microRNAs (miRNAs) target the pro-apoptotic protein Bim, leading to its decreased expression and promoting cell survival (Marquitz et al., 2011). EBV miR-BART5 can further trigger the inhibition of apoptosis by targeting a cellular protein named p53 upregulated modulator of apoptosis (PUMA) (Choy et al., 2008b).

Additionally, v-miRNAs can also promote latency by targeting host genes. The v-miRNAs originating from KSHV can target various host mRNAs, among which is the IkBa mRNA. This targeting results in the activation of NF-kB signaling and serves to prevent viral lytic replication (Lei et al., 2010). Likewise, it can be observed that the v-miRNA originating from the EBV’s BART18 element is capable of targeting the mRNA of MAP kinase 2 (MAP3K2). Through this targeting, the initiation of lytic viral replication is effectively prevented (Qiu and Thorley-Lawson, 2014).

Several v-miRNAs have been observed to promote viral survival by targeting genes that are involved in the antiviral immune response. Viral miRNAs can interfere with the recognition and targeting of effector cells, including T cells and NK cells, by directly inhibiting them or indirectly modulating the expression of cytokines such as IL-6. For instance, KSHV miR-K12-9 and miR-K12-5 target two distinct points of the TLR/Interleukin-1R signaling cascade (IRAK1 and MYD88), thereby reducing the expression of inflammatory cytokine mRNAs (Abend et al., 2012). Additionally, KSHV miR-K12-11 and miR-K12-5 expressed by KSHV obstruct the recognition of infected cells by natural killer (NK) cells by directly targeting the activation-induced cytidine deaminase (AID) coding gene (Bekerman et al., 2013). Similarly, v-miRNAs expressed by HCMV are known to facilitate evasion from the NK antiviral response. HCMV-expressed miR-UL112-1, miR-US5-1, and miR-US5-2 have been observed to regulate the mRNAs that encode IL6 and TNF-a, two cytokines that are associated with the secretory pathways. This regulatory effect alters the secretion of host cytokines and results in the obstruction of the antiviral response (Hook et al., 2014).

Despite the previous mention of a limited understanding regarding the functions of v-miRNAs encoded by RNA viruses, they seem to preserve the functions of v-miRNAs that have been described in DNA viruses. Indeed, v-miRNAs originating from RNA viruses have been associated with apoptosis and tumorigenesis processes through the regulation of host mRNAs. For example, HIV-1 encoded Tar-miR-5p and Tar-miR-3p have been shown to downregulate ERCC1 and IER3, host genes vital for cell survival and apoptosis, thus allowing for HIV-1 infected cells to survive for longer periods by preventing host cell death (Klase et al., 2009). Moreover, BLV and SFV-encoded miRNAs mimic host miRNAs and are involved in similar processes. SFV miR-S4-3p mimics the seed sequence of cellular miR-155, which target genes involved in cell proliferation (Kincaid et al., 2014). Similarly, BLV-miR-B4 shares partial sequence identity and shared common targets with the host miRNA, miR-29, that have been associated with B-cell neoplasms, suggesting that BLV-miR-B4 may contribute to BLV-induced tumorigenesis (Kincaid et al., 2012).

The extent to which v-miRNAs contribute to SARS-CoV-2 infection remains largely unexplored since most of the identified v-miRNAs have been predicted solely through computational analyses. Nevertheless, some insights have been gained in this area. In fact, the initial discovery of v-miRNAs in SARS-CoV in 2017 revealed that they possess the capacity to induce the release of pro-inflammatory cytokines and contribute to the inflammatory pulmonary pathology that is characteristic of SARS (Morales et al., 2017). In a more recent study, four microRNA-like molecules with unique characteristics were discovered, and two of these, SCV2-miR-ORF1ab-1-3p and SCV2-miR-ORF1ab-2-5p, were shown to play a crucial role in evading the type I interferon response by targeting several genes in this signaling pathway (Zhu et al., 2021). Two independent studies also showed that SARS-CoV-2 v-miRNAs derived from a conserved stem-loop structure of the ORF-7a transcript are associated with the regulation of interferon signaling (Pawlica et al., 2021; Singh et al., 2022).

In summary, v-miRNAs play a crucial role in complex virus-host interactions. By promoting the switch between latency and lytic replication, supporting persistent infection and immune escape, and targeting host genes involved in various cellular processes, v-miRNAs contribute to the pathogenesis of viral infections, including the ones caused by RNA viruses. While much is still unknown about these molecules, it is clear that their study will provide important insights into the intricate interactions between RNA viruses and their hosts.

## 4 Other classes of viral noncoding RNAs

In addition to v-miRNAs, other species of viral-encoded regulatory RNAs have been described. Similar to v-miRNAs, these ncRNAs are mainly encoded by DNA viruses, particularly those in the family Herpesviridae. Even though miRNAs and long noncoding RNAs have received increased attention in recent decades, studies of other classes of non-coding RNAs originating from viruses have been scarce. The difficulty in detecting these molecules within viral genomes and separating them from degradation products may be the reason for this. In addition, it is quite probable that some molecules identified as non-canonical miRNAs may belong to different classes of ncRNAs due to an excessive focus on their size, thereby failing to perform appropriate functional validation. Despite these limitations, some studies have demonstrated that RNA viruses can produce functional ncRNA molecules of medium to small sizes in addition to miRNAs (Table 3).

**TABLE 3:** RNA viruses-encoded sncRNAs aside from miRNAs.

Virus	v-sncRNA	Function	References
**Flaviviridae**			
All members of *Flavivirus*genus	sfRNAs	Modulation of virus transmission; inhibition of inflammatory gene expression	Pijlman et al. (2008), Funk et al. (2010)
			Akiyama et al. (2016), Michalski et al. (2019), Slonchak et al. (2022)
			Pompon et al. (2017), Yeh et al. (2022)
**Rhabdoviridae**			
VSV	leRNAs	Control viral replication	Wilusz et al. (1983)
RABV			Kurilla et al. (1984), Zhang et al. (2017)
**Paramyxoviridae**			
SeV	leRNAs	Control viral replication	Leppert et al. (1979)
**Orthomyxoviridae**			
IAV	leRNAs	Regulate the switch from mRNA synthesis to viral genome replication	Perez et al., 2010 (2012), Umbach et al. (2010)
**Kolmioviridae**			
HDV	ribozyme RNA	Ribozyme activity; Promote viral replication	Been and Wickham (1997)
**Retroviridae**			
HIV	siRNA	Restriction of replication	Bennasser et al. (2005)
SIV		Unknown	

Summary of known sncRNAs encoded by RNA viruses (excluding miRNAs) and their functions. Besides miRNAs, RNA viruses encode other classes of regulatory small RNA such as flavivirus-encoded sfRNAs, leRNAs encoded by Rhabdoviridae, Paramyxoviridae and Orthomyxoviridae families, a ribozyme like RNA found in HDV genome and retrovirus-encoded siRNAs. VSV, Vesicular stomatitis virus; RABV, Rabies virus; SenV, Murine respirovirus (formerly Sendai virus); IAV, Influenza A virus; HDV, Hepatitis delta virus; HIV: HIV-1, Human immunodeficiency virus-1; SIV, Simian immunodeficiency virus.

### 4.1 Subgenomic flavivirus RNA

Flaviviruses have in common the ability to produce small non-coding RNAs known as subgenomic flavivirus RNAs (sfRNAs), which are involved in immune evasion. When XRN1, a major component of the mRNA decay pathway, engages the viral genome, small fragments of RNA are produced originating sfRNAs as a result of the incomplete degradation of the viral genome (Chapman et al., 2014). Moreover, the presence of pseudoknot secondary structure in the 3′UTR of flavivirus genomes protects the genomic RNA from being totally degraded (Akiyama et al., 2016). As a result, these structures have been used as markers for the identification of sfRNAs conserved in all members of the flavivirus genus, such as West Nile viruses (WNV), Dengue Virus (DENV), and Zika virus (Chapman et al., 2014; Akiyama et al., 2016).

Several studies elucidated the RNA structural elements required for XRN1 activity and demonstrated the role of sfRNAs in viral evasion of the type I IFN-mediated antiviral response in vertebrates and the Toll pathway in mosquitoes (Schnettler et al., 2012; Slonchak and Khromykh, 2018; Slonchak et al., 2022). Among these viruses, Zika produces two molecules, sfRNA1, and sfRNA2 that target the STAT1 pathway leading to type I interferon 2 (IFN2) inhibition and facilitating viral pathogenesis (Slonchak et al., 2022). The same study also showed that sfRNA modulates the STAT1 pathway by binding and stabilizing NS5 viral protein highlighting the importance of RNA-protein interaction in the process of immune evasion (Slonchak et al., 2022). Moreover, mutant WNV lacking production of sfRNA1, or sfRNA1 and sfRNA2 exhibit reduced viral growth and pathogenicity in human cells in culture and mice (Pijlman et al., 2008) and DENV-encoded sfRNAs have been shown to colocalize and interact with targets previously implicated in modulating viral infection (Bidet et al., 2014). Later studies demonstrated the pivotal role of sfRNA in the replication, dissemination, and transmission of flaviviruses as well as their participation in signaling and apoptosis, a common feature between sfRNAs and v-miRNAs (Reviewed in (Slonchak and Khromykh, 2018)).

### 4.2 RNA virus encoded-leRNA

Small viral leader RNAs (leRNAs) are an often overlooked class of small regulatory RNAs originated in the 5′end of the viral genomic RNA. The precise size of these molecules has been subject to some debate. LeRNAs were first identified in negative-strand RNA viruses (rabies virus and vesicular stomatitis virus) with sizes greater than 40 nucleotides (Wilusz et al., 1983; Kurilla et al., 1984), however, more recent analyses of influenza virus genomes have revealed the presence of leRNAs that have a size between 18 and 27 nucleotides, similar to miRNAs (Perez et al., 2010; Umbach et al., 2010). This small RNA is distinguished from other classes of sncRNAs, namely, miRNAs, by the relatively heterogeneous length and, most importantly, by the fact that it retains a 5′triphosphate, which prevents it from being incorporated into the RISC complex (Umbach et al., 2010).

The most studied leRNAs are the vesicular stomatitis virus (VSV) leRNAs which were first identified over 40 years ago (Wilusz et al., 1983). These molecules range from 45 to 48 nucleotides and have been suggested to play a vital role in the VSV life cycle by regulating the switch from mRNA transcription to genomic RNA replication (Wilusz et al., 1983). Moreover, VSV leRNAs as well as Rabies virus (RABV) leRNAs interact with protein La (Wilusz et al., 1983; Kurilla et al., 1984), which may shield leRNA from RIG-1, a known activator of IFN gene expression (Bitko et al., 2008). Moreover, a recent study also demonstrated that RABV leRNA may inhibit viral replication by interaction with the Hsc70 host protein (Zhang et al., 2017). In the same year, two studies demonstrated that the influenza virus encodes leRNAs of similar size to miRNAs (Perez et al., 2010; Umbach et al., 2010). These molecules appear to regulate the switch from transcription to replication in a similar manner to the SVS and RABV leRNAs (Perez et al., 2010). Interestingly, both studies report different percentages of leRNAs detected, which can be attributed to the different RNA sequencing protocols used.

Due to their similar size and function, it is possible that approaches used in computational prediction of miRNAs could miss-identify leRNAs as belonging to this class. The authors of a work that compared deep learning-based approaches for predicting sncRNA classes found that the use of specific features can increase misprediction rates of leRNAs as miRNAs (Chantsalnyam et al., 2021). Together, these observations may call into question the interpretation of many miRNA-like molecules identified in the literature and available databases.

### 4.3 Putative siRNAs in HIV-1, HIV-2, and SIV

The small interfering RNAs (siRNAs) are regulatory RNAs with a similar size range to miRNAs. These molecules result from the direct cleavage of long double-stranded RNAs by Dicer, and they are responsible for regulating gene expression by binding to complementary sites within mRNAs, thus preventing translation (Nozawa and Kinjo, 2016). In addition, viral siRNAs have a similar structure to host endogenous siRNAs, with monophosphate groups at the 5′and 2′-O-methyl groups at the 3′ends (Ding, 2010).

*D. melanogaster* and plants were the first organisms to show evidence of virally encoded siRNAs (Ding, 2010; Qu, 2010; Sabin et al., 2010). It was shown that infection of *Drosophila* with RNA viruses induces DICER2-dependent production of siRNAs with average size of 21 nucleotides (Sabin et al., 2010). Further, they showed that these siRNAs select and destroy their mRNA targets by guiding the slicing of the target mRNA by an Argonaute (AGO) protein (Sabin et al., 2010). It is our understanding that only one article, at the time of this review, proposed that viral siRNAs were produced in infected animal cells. This work identified five putative siRNAs encoded by HIV-1, HIV-2, and SIV, which were processed in a DICER-dependent manner and exhibited virus-restricting properties (Bennasser et al., 2005).

### 4.4 Hepatitis delta virus ribozyme RNA

Hepatitis Delta Virus (HDV) is an RNA virus that encodes a self-cleaving sequence that contributes to viral replication and is considered to function as a ribozyme *in vivo* (Been and Wickham, 1997). The ribozyme-like molecule is about 85 nucleotides long and adopts a secondary structure with four paired regions (P1–P4). However, the functional role of this v-sncRNA in infection is still unknown and further studies would be necessary to clarify the classification of this molecule in a specific sncRNAs class (Been and Wickham, 1997).

## 5 Potential therapeutic approaches for v-miRNAs: lessons from COVID-19 pandemic

The global challenge of combating viral diseases and virus-associated cancers remains an ongoing health concern. Small non-coding RNAs, including miRNAs, have been the focus of intense research over the past decade due to their significant regulatory roles in gene expression. As reviewed in the previous sections, v-miRNAs have been identified in several DNA and RNA viruses and present the ability to control both viral and host gene expression. Some v-miRNAs have been shown to play a vital role in the viral life cycle, including viral replication and persistence in host cells.

Given the limited availability of treatments for the majority of emerging viruses, miRNA-based therapeutics are being recognized as a new category of drugs with the potential to combat a wide range of diseases, including infectious diseases. The safe and effective delivery of adequate amounts of miRNAs to infected areas represents a significant hurdle for the clinical application of miRNA-based therapies (Chakraborty et al., 2017). Due to the involvement of host miRNAs in a broad range of cellular functions, sustained treatment with antagomirs may result in undesirable side effects associated with the dysregulation of target gene expression. Consequently, as it reduces the danger of side effects and off-targets, targeting viral miRNAs may offer a more viable therapeutic strategy. One common approach for miRNA-based therapies is the use of chemically modified oligonucleotides. One of the earliest demonstrations of oligonucleotide chemical modification for the inhibition of miRNA activity involved the development of antagomirs (sequence-specific miRNA inhibitors). Some studies have been applying the use of antagomir to study v-miRNAs functions. For instance, the use of a specific antagonism of KSHV miRK9 in latently infected cells enhanced the frequency of spontaneous lytic reactivation and allowed to determine the role of this v-miRNA in promoting latency (Bellare and Ganem, 2009). Moreover, another work used antagomirs to identify candidate targets of KSHV miRNAs and showed that transfection of latently infected BCBL-1 cells with antagomirs to miR-K5 increased the levels of BCLAF1, an apoptosis promoter (Ziegelbauer et al., 2009). Although these studies do not focus on the development of therapeutic approaches, they illustrate the possibility of using miRNA-based therapies to downregulate v-miRNAs involved in different processes and highlight the importance of considering v-miRNAs as possible therapeutic targets.

One of the fields that have most contributed to the investigation of miRNAs as therapeutic targets is the study of virus-induced tumors. Significant strides have already been made in the pursuit of targeting cellular miRNAs as potential anti-cancer therapeutic agents, and even as innovative drugs. Conversely, the targeting of v-miRNA in tumorigenesis represents a novel approach (Hull et al., 2022). Currently, a cellular miR-122 inhibitor is in a phase II clinical trial (Janssen et al., 2013). This liver-specific miRNA is involved in HCV infection, and its inhibitor shows promising effects against infection (Janssen et al., 2013). Given its success, similar antagonist mechanisms could be explored in the targeting of v-miRNAs. An additional study involving EBV-miRBART7-3p showed the delivery of an antagomir for this v-miRNA using gold nanoparticles successfully inhibited this v-miRNA, ultimately suppressing tumor formation in mice (Cai et al., 2015). Moreover, the use of miRNA sponges has also been suggested as a successful mechanism to target EBV miRNAs and silence specific genes in EBV-infected cells (Choy et al., 2008a). A recent study also developed a novel strategy to deliver anti-miRNA targeting KSHV miRNAs (Ju et al., 2020). This work reported that locked nucleic acid (LNA) delivered by carbon dots (Cdots) can be employed for specific knockdown of KSHV miR-K1, miR-K4, and miR-K11 to induce apoptosis and suppress the cell proliferation of KSHV-positive cells (Ju et al., 2020). Taken together, these studies demonstrate that miRNA-sponges and anti-miRNA oligonucleotide therapeutics may represent viable approaches for targeting specific viral miRNAs that contribute significantly to the development of virus-associated tumors.

Another potential approach based on targeting v-miRNAs is the use of CRISPR-Cas9 gene editing technology to target and degrade v-sncRNAs. Over the past years, the use of CRISPR/Cas9 as a powerful and user-friendly gene-editing method has significantly grown and now encompasses virtually all areas of biomedical research, including investigations into human viruses. Initially, this technology was implemented to obstruct protein expression, but with the progress of technology, new protocols have been devised that can effectively target host miRNAs (Chang et al., 2016; Yang et al., 2018; Godden et al., 2022). While this technique is primarily used to target host genome components, current evidence suggests that it can also be leveraged to hinder the activity of miRNAs originating from viruses. A recent study showed that by using CRISPR/Cas9 to downregulate KSHV miRNAs it was possible to alter the expression of v-miRNAs downstream target genes and KSHV lytic genes in host PEL cells. The ablation of v-miRNAs in KSHV-infected cells leads to the upregulation of lytic genes but not latent genes showing that this strategy can be used to reactivate the viral genome *in vitro* (Liang et al., 2020). Additionally, another study described a similar CRISPR/Cas9-based strategy to efficiently mutate miRNAs encoded by Marek’s disease virus. The authors used a plasmid-based system to introduce specific mutations into the miRNAs and demonstrated deletion of specific miRNAs clusters or individual miRNAs can produce distinct effects either promoting or inhibiting viral replication (Luo et al., 2020).

Adding to the therapeutic perspective, miRNAs, including v-miRNAs, have clinical relevance as biomarkers due to their potential secretion in body fluids such as blood, saliva, and urine. A recent study on the EBV v-miRNA BARTT7 showed that this molecule can be an excellent tool to diagnose EBV-associated nasopharyngeal carcinoma and assess treatment effectiveness (Lu et al., 2020). Likewise, HCMV-encoded miR-US4-1 is used to predict the efficacy of IFN-α treatment in hepatitis B (Pan et al., 2016). RNA virus-encoded v-miRNAs have also been reported as potential biomarkers. EBOV miR-VP-3p was found in the serum of Ebola disease patients before development of viremia with detectable Ebola genomic RNA, suggesting that this v-miRNA can be used as an early biomarker of Ebola disease and possibly serve as a tool to predict future Ebola outbreaks (Chen et al., 2016). Interestingly, the same strategy was used in the fight against COVID-19. SARS-CoV-2 encoded miR-nsp3-3p was monitored in patients progressing from mild/moderate to severe symptoms and was detected in patients’ serum at the pre-severe stage. This v-miRNA serves as a biomarker that helps define priority patients based on their risk of developing severe disease (Fu et al., 2021).

The utilization of nanoparticles in the oral delivery of antagomirs or miRNAs presents a promising therapeutic strategy against viral infections, although currently still in its developmental stage and with a lack of studies pertaining to v-miRNAs. These miRNA nano-therapeutics offer several advantages, including high specificity to infection sites and minimal off-target effects. Various nano formulations have been proposed in the literature for efficient oral delivery of miRNAs (Martirosyan et al., 2014; Arntz et al., 2015; Kanwar et al., 2015; Beuzelin et al., 2019; Deng et al., 2019; Zou et al., 2019), and their use could be an efficient v-miRNA-based strategy for future approaches.

The COVID-19 pandemic has provided a unique opportunity to deepen our understanding of viral-encoded miRNAs and their potential as targets for the development of new therapies for RNA virus infections. However, although several studies have predicted possible v-miRNAs, identified possible targets, and highlighted their therapeutic relevance (Morales et al., 2017; Saini et al., 2020; Aydemir et al., 2021; Liu et al., 2021; Meng et al., 2021; Pawlica et al., 2021), few have proposed actual therapeutic approaches based on these viral molecules. In addition to the previously mentioned work that identified miR-nsp3-3p as a potential biomarker to distinguish patients developing severe COVID-19 from mild/moderate infections (Fu et al., 2021), we are aware of only another study that proposed a therapeutic strategy based on v-miRNAs, actually referring to the svRNA-N SARS-COV encoded molecule, implementing an antagomir-based strategy to inhibit its expression (Morales et al., 2017). When mice were inoculated with locked nucleic acids (LNA) corresponding to anti-svRNA-N, they demonstrated reduced pulmonary inflammation and pro-inflammatory cytokine production, although there was no increase in survival (Morales et al., 2017).

In conclusion, targeting viral miRNAs represents a promising strategy for the development of new therapeutic approaches against viral diseases and virus-associated cancers. Chemical modification of oligonucleotides, miRNA-sponges, and CRISPR/Cas9 gene editing technology are among the methods currently being explored for the effective inhibition of v-miRNAs. Notwithstanding, further investigation is needed to enhance our knowledge of v-miRNAs as well as to provide new therapeutically relevant insights.

## 6 Final remarks and unsolved questions

In this review, we have explored the diverse functions of sncRNAs in viral infection, with a particular focus on v-miRNAs derived from RNA viruses. While previous reviews have been published on this topic, most do not cover the full range of sncRNAs encoded by RNA viruses, and some only focus on DNA viruses, or a specific class of viral sncRNAs such as miRNAs. Furthermore, there is a lack of up-to-date reviews on these molecules in the wake of the COVID-19 pandemic. Given the recent developments spurred by an intense research focus on the SARS-CoV-2 virus, we felt it was relevant to revisit the existing knowledge about sncRNAs produced by RNA viruses.

Despite their potentially critical role in virus-host interactions, research on miRNAs in RNA viruses has received limited attention, highlighting the need for further investigation. This has been mostly due to two major fallen premises: that the presence of precursor miRNA hairpins would lead to the destructive cleavage of viral genomes; and that the fact that most RNA viruses undergo cytoplasmic replication would prevent access to the miRNA biogenesis machinery. The clear demonstration of non-canonical biogenesis pathways for viral-derived miR-like molecules has subverted this reasoning (see, for example, Kincaid et al., 2012 or Hussain et al., 2012). Another key point that has been put forth against the relevance of v-miRNAs as regulators of RNA virus replication is related to the fact that these molecules seem to be particularly relevant for the establishment of persistent infections, which are a hallmark of DNA viruses (although also typical of retroviruses) (Aguado and tenOever, 2018). The increased number of epidemic and pandemic outbreaks caused by RNA viruses in the past decade has uncovered a new paradigm suggesting that RNA virus persistence may be an underappreciated phenomenon (Griffin, 2022). In this scenario, the multiple observations regarding the existence of RNA virus derived v-miRNAs that reduce the expression of viral transcripts or interfere with host cell processes and immune system acquire increasing relevance. The fact that these v-miRNAs may be involved in processes other than acute infection and replication also helps to undermine the fourth argument against the biological relevance of many of the molecules identified so far: the fact that they do not accumulate to significant levels in RNA-seq datasets derived from bulk-sequencing of infected cells.

In recent years, the intense study of SARS-CoV-2 as a consequence of the COVID-19 pandemic has underscored the limitations in our understanding of RNA virus encoded sncRNA molecules and their critical importance in understanding the interplay between host cells and virus during infection. The intense focus on SARS-CoV-2 has led to rapid developments in the field, including the discovery of a number of v-miRNAs that are thought to play key roles in its pathogenesis and virulence. These findings have provided valuable insights into viral molecular mechanisms and pave the way for developing new therapies and diagnostic tools for viral infections. While the functions of sncRNAs are varied and complex, they also represent a promising target for therapeutic intervention. By targeting v-miRNAs, it may be possible to disrupt key aspects of viral replication and transmission, developing novel treatments for viral infections and virus-related diseases. Moreover, v-miRNA-based therapeutics may offer several advantages over traditional antiviral drugs, including greater specificity and a lower risk of off-target effects.

Finally, it is important to note that the intense production of literature regarding sncRNAs and SARS-CoV-2 has also highlighted the pitfalls of many investigations into the topic. First and foremost, research relying solely on computational methods for identifying candidate miRNAs and predicting their targets has created an abundance of mostly irrelevant information that does not help the field move forward. While these methods can be useful for generating hypotheses, experimental validation is critical to confirm the expression and function of v-miRNAs in viral infections. Given the non-canonical nature of v-miRNA molecules and the difficulties in working with natural infection models, the definition of what constitutes a *bona fide* proof-of-function needs to be discussed in detail by the research community. Furthermore, the need for a comprehensive and centralized database describing v-miRNAs is paramount. The systematic analysis we performed of the three available repositories revealed many insufficiencies that constitute significant challenges for researchers interested in studying these molecules, including the lack of distinction between validated and computationally predicted molecules.

The study of viral sncRNAs and v-miRNAs is a rapidly evolving field, with many open questions and challenges remaining. Key areas for future research include the development of more precise methods for characterizing sncRNA expression and function, the elucidation of the mechanisms by which they are produced and processed in infected cells, and the exploration of their role in regulating viral and host processes. Overall, we believe this to be an exciting and promising area of inquiry, with the potential to yield significant insights into the complex dynamics of viral infections and host-virus interactions. By continuing to explore the multifaceted roles of sncRNAs in different viral systems, we may be able to develop new strategies for preventing and treating viral diseases, and ultimately improve global health outcomes.
